# Fluorescent Nanocable as a Biomedical Tool: Intracellular Self-Assembly Formed by a Natural Product Interconnects and Synchronizes Mitochondria

**DOI:** 10.1021/acsnano.4c06186

**Published:** 2024-07-31

**Authors:** Xueqian Zhao, Fei Wang, Chuen Kam, Ming-Yu Wu, Jianyu Zhang, Changhuo Xu, Kai Bao, Qiyuan He, Ruquan Ye, Ben Zhong Tang, Sijie Chen

**Affiliations:** † School of Life Sciences, The Chinese University of Hong Kong, Hong Kong 999077, China; ‡ Ming Wai Lau Centre for Reparative Medicine, Karolinska Institutet, Hong Kong 999077, China; § Department of Chemistry, Hong Kong Branch of Chinese National Engineering Research Center for Tissue Restoration and Reconstruction, and Guangdong-Hong Kong-Macau Joint Laboratory of Optoelectronic and Magnetic Functional Materials, The Hong Kong University of Science and Technology, Hong Kong 999077, China; ∥ Ministry of Education Frontiers Science Center for Precision Oncology, Faculty of Health Sciences, University of Macau, Macao 999078, China; ⊥ Department of Materials Science and Engineering, Department of Chemistry, City University of Hong Kong, Hong Kong 999077, China; # School of Science and Engineering, Shenzhen Institute of Aggregate Science and Technology, The Chinese University of Hong Kong, Shenzhen (CUHK-Shenzhen), Guangdong 518172, China

**Keywords:** intracellular assembly, nanofiber, fluorescent probe, mitochondrial network, mitochondrial membrane potential oscillations

## Abstract

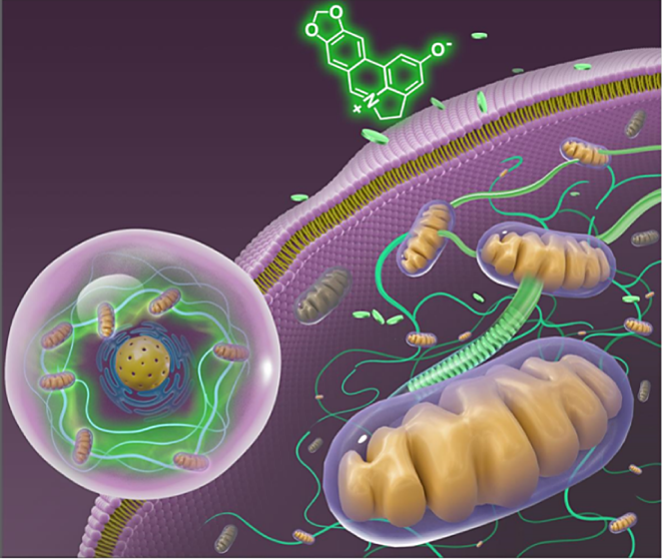

Self-assembly processes commonly occur in various biological contexts to form functional biological structures. However, the self-assembly of nanofibers within cells by heterologous molecules showing a biological function is rare. In this work, we reported the intracellular formation of fluorescent nanofibers by a natural small molecule, lycobetaine (LBT), which facilitated the direct physical connection between mitochondria and synchronized their membrane potential oscillations. The luminescent properties of LBT enabled the real-time observation of nanofiber formation, while the semiconductive nature of the LBT nanofiber facilitated electrical signal transduction among the connected mitochondria. This study introduces an approach to modulate mitochondrial connectivity within cells using “nano-cables” which facilitate studies on synchronized mitochondrial operations and the underlying mechanisms of drug action.

Mitochondria, being principal energy-generating organelles in eukaryotic cells, perform crucial roles in apoptosis, lipid metabolism, biosynthesis, and respiratory ATP production.^1^ In the process of respiration, mitochondrial membrane potential (ΔΨ_m_)^2^ is a dynamic parameter that can be evaluated by membrane potential-sensitive fluorescent dyes.^3,4^ Although fluctuates from time to time, the ΔΨ_m_ holds a stable homeostasis with the steady state of about −180 mV tolerating variations from −160 to −220 mV.^5,6^ Nonetheless, subtle deviations in the stable levels of the ΔΨ_m_ can significantly impact mitochondrial functions, potentially leading to mitochondrial dysfunction.^7^ The decline in ΔΨ_m_ levels serves as an indicator of cell disorders, which can subsequently trigger autophagy,^8^ apoptosis,^9^ and necrosis.^10^ The loss of ΔΨ_m_ has been linked to various diseases, including keratitis,^11^ diabetes,^12^ neurodegenerative diseases,^13^ and cancer.^14^ On the other hand, abnormally elevated levels of ΔΨ_m_ can contribute to the development of severe conditions, including glaucoma and frontotemporal dementia.^15^ Therefore, it holds great significance to study ΔΨ_m_ abnormality in both biological studies and the diagnosis of associated diseases. Much effort has been focused on monitoring and regulation at the level of individual mitochondria. However, the propagation of ion signals with ΔΨ_m_ oscillations among interconnected mitochondria, particularly how individual mitochondria communicate with one another, remains obscure in cell-based studies. The linkage of the individual mitochondria by a conductive “nano-cable” may synchronize the ΔΨ_m_ oscillations throughout the entire mitochondrial network and provide us with an opportunity to study the wave propagation at a cellular level.^16^ However, the realization of such a system has not yet been achieved.^17−19^

One possible method to construct such a “nano-cable” is through self-assembly. The self-assembly of monomers is commonly found in nature and living beings.^20^ The cytoskeleton is formed from protein components that create a dynamic network of filaments within the cell for metabolism, proliferation, and intracellular transport.^21,22^ The development of an intracellular self-assembly system with high precision and multiple functions has gained increasing attention.^23−25^ A series of synthetic nanomaterials, including but not limited to polymers, peptides, and polyaromatic materials, have been created for intracellular assembly in the past two decades.^26−28^ The exogenous intracellular assembly system with orders and dynamics can be functional in stimuli-responsiveness,^26^ therapy,^29,30^ catalysis,^31,32^ and imaging.^33−37^ Presumably, the intracellular synthesis of these artificial fibers through a bottom-up approach can interconnect organelles for signal transduction, thereby revealing cellular processes under diverse physiological conditions.^38−41^ However, intracellular self-assembly processes pose an inherent challenge due to the highly dynamic and crowded cellular environment, which makes achieving optimal scales and defined intracellular targets difficult.

Lycobetaine (LBT), a natural betaine-type alkaloid discovered in some Amaryllidaceae plants, such as *Lycoris radiata*, has been reported as an antineoplastic drug.^42−44^ However, its intrinsic optical properties, similar to other natural products,^45−50^ are often overlooked. In this work, we systematically investigate the rich photophysical properties of LBT in detail, including its pH-responsive fluorescence behavior and emission mechanism in both solution and aggregate states. The real-time monitoring of the formation of LBT nanofibers *in situ* is also illustrated. Assisted by a ΔΨ_m_ probe, we observed synchronized ΔΨ_m_ oscillations of LBT-nanofiber-connected mitochondria in live cells. Our research indicates that the oscillations in mitochondrial membrane potential are contingent on the K^+^ concentration gradient. Consequently, LBT nanofibers, which facilitate interconnection between mitochondria, offer a tool for advancing mitochondrial research.

## Results and Discussion

### Photophysical Properties of LBT

**Figure 1:**
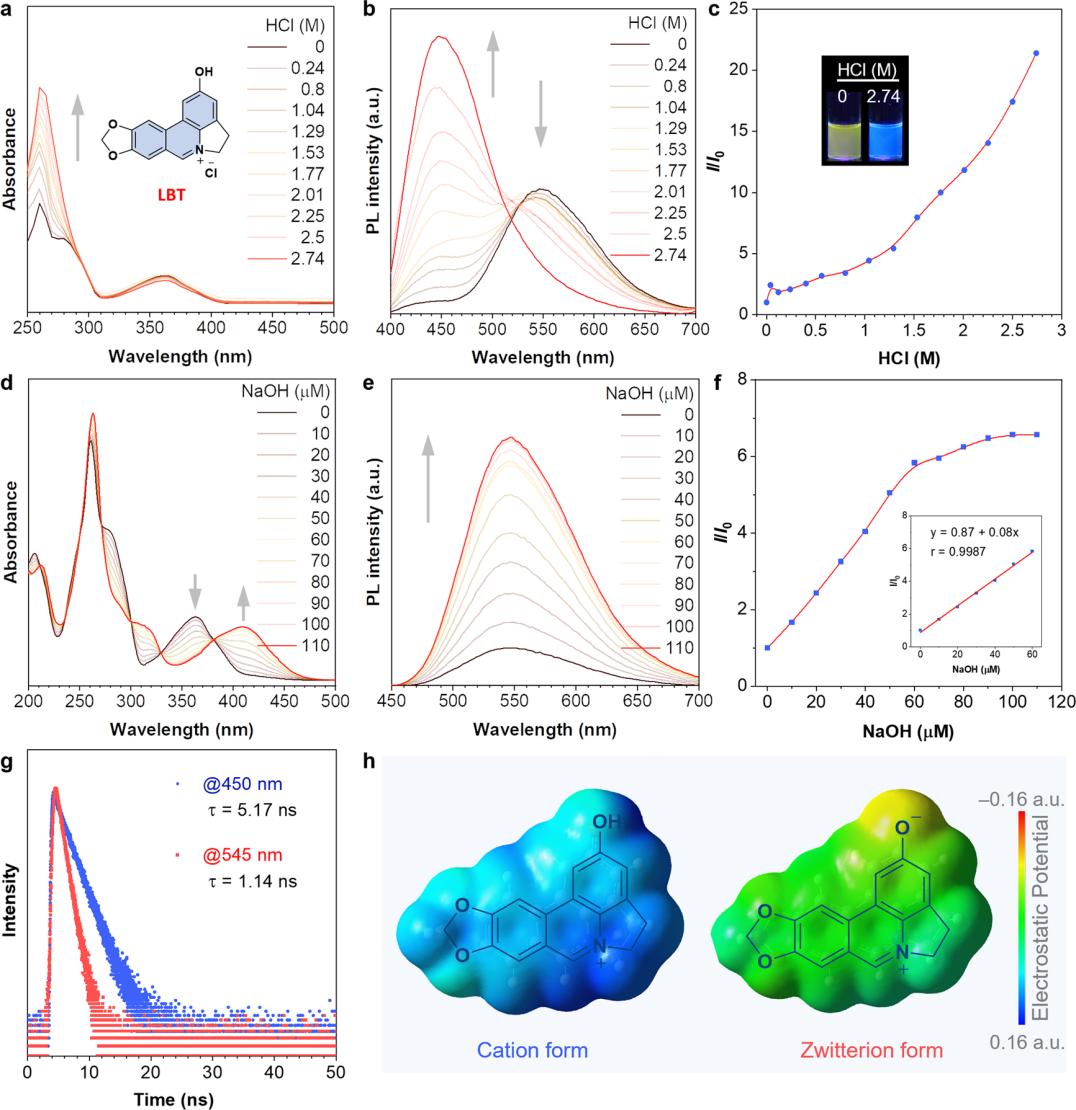
Photophysical properties of LBT in aqueous solutions. (**a)** Absorption spectra and **(b**) emission spectra (λ_ex_ = 365 nm) of LBT at different HCl concentrations. The inset shows the chemical structure of the LBT. (**c**) Relative PL intensity changes of LBT at 450 nm. Inserts show the corresponding PL images. *I*_*0*_ is the initial intensity at 450 nm. λ_ex_ = 365 nm. (**d**) Absorption spectra and (**e**) emission spectra (λ_ex_ = 420 nm) of LBT at different NaOH concentrations. (**f**) Relative PL intensity changes in LBT at 545 nm. The inset shows the linear relationship between PL intensity changes and NaOH concentrations. *I*_*0*_ is the initial intensity at 545 nm. λ_ex_ = 420 nm. (**g**) Time-resolved PL decay curves of LBT at room temperature. (**h**) The structures of cation and zwitterion forms of LBT and their corresponding electrostatic potential maps calculated by using the M06-2X density functional and 6-31+G (d, p) basis set.

LBT is a π-conjugated molecule featuring a hydroxyl group, rendering it a prospective pH-responsive species.^51−53^ Thus, we initially examined the photophysical characteristics of LBT in an aqueous solution with the addition of hydrochloric acid (HCl) or sodium hydroxide (NaOH). The ultraviolet–visible (UV–vis) spectra of LBT were subsequently recorded (Figure 1a). With increasing concentrations of HCl, the absorption band spanning 250 to 300 nm exhibited heightened intensity and sharper features, while the absorption band ranging from 320 to 420 nm remained relatively unchanged. Under excitation at 365 nm, a pronounced enhancement of the emission peak at 450 nm was observed (Figure 1b), accompanied by a gradual decrease in fluorescence at 545 nm upon the addition of HCl. In comparison to the solution without HCl, the fluorescence intensity of LBT at 450 nm exhibited an ∼21-fold increase, accompanied by a color transition from yellow to blue in the presence of 2.74 M HCl (Figure 1c). This indicates a pronounced protonation of LBT in highly acidic conditions. Conversely, the introduction of an aqueous NaOH solution induced a gradual emergence of the absorption band spanning 350 to 450 nm (Figure 1d). Simultaneously, the emission peak at 545 nm displayed a linear increase upon excitation at 420 nm (Figures 1e,f), indicating the deprotonation of LBT under alkaline conditions. Specifically, its emission intensity at a wavelength of 545 nm displayed an inverse trend when excited at different wavelengths in Britton–Robinson (B–R) buffer solutions within the pH range from 2.0 to 11.0 (Figure S1). To further investigate this phenomenon, we examined the excitation spectra. As shown in Figure S2, the excitation maxima were determined to be 368 and 403 nm, corresponding to the emission maxima of 450 and 545 nm, respectively. The corresponding emission lifetimes were measured to be 5.17 and 1.14 ns, indicating the nature of fluorescence emission (Figure 1g). These findings suggest that the emission arises from two distinct excited states. These results support the assertion that the protonation (cationic form) and deprotonation (zwitterionic form) of LBT occur in response to pH changes.

To further verify that the distinct emission properties of the probes at different pH originate from the cationic and zwitterionic forms, we conducted calculations and obtained density functional theory (DFT)-molecular electrostatic potential maps. By comparing the electron density distributions of the two forms, we sought to establish their correlations with the observed photoluminescence (PL) characteristics (Figure 1h).^54^ The *N*-cationic regions contain the lowest electron density and dominate the charges of the entire cationic form of the molecule. Meanwhile, the *O*-anionic regions, characterized by their high charge density, are prominent in the zwitterionic form. Overall, the charge density of the zwitterionic form is significantly higher than that of the cationic form. The proton nuclear magnetic resonance (^1^H NMR) spectrum of LBT was obtained under conditions of both protonation (treated with HCl) and deprotonation (treated with NaOH) as shown in Figure S3. Upon deprotonation, the proton signals of LBT exhibited a high-field shift relative to those in the protonated state. This observation is in agreement with the theoretically calculated electronic environment. Next, the highest occupied molecular orbital (HOMO) and the lowest unoccupied molecular orbital (LUMO) plots were determined by DFT calculations (Figure S4). The corresponding energy gaps are calculated to be 5.60 and 4.36 eV for cationic and zwitterionic forms, respectively, which is consistent with the measurement of absorption spectra. Therefore, these results confirm that cationic and zwitterionic forms of LBT are in charge of the diverse luminescence in the solution state.^55^

### LBT Self-Assembles to Form Nanofibers

**Figure 2:**
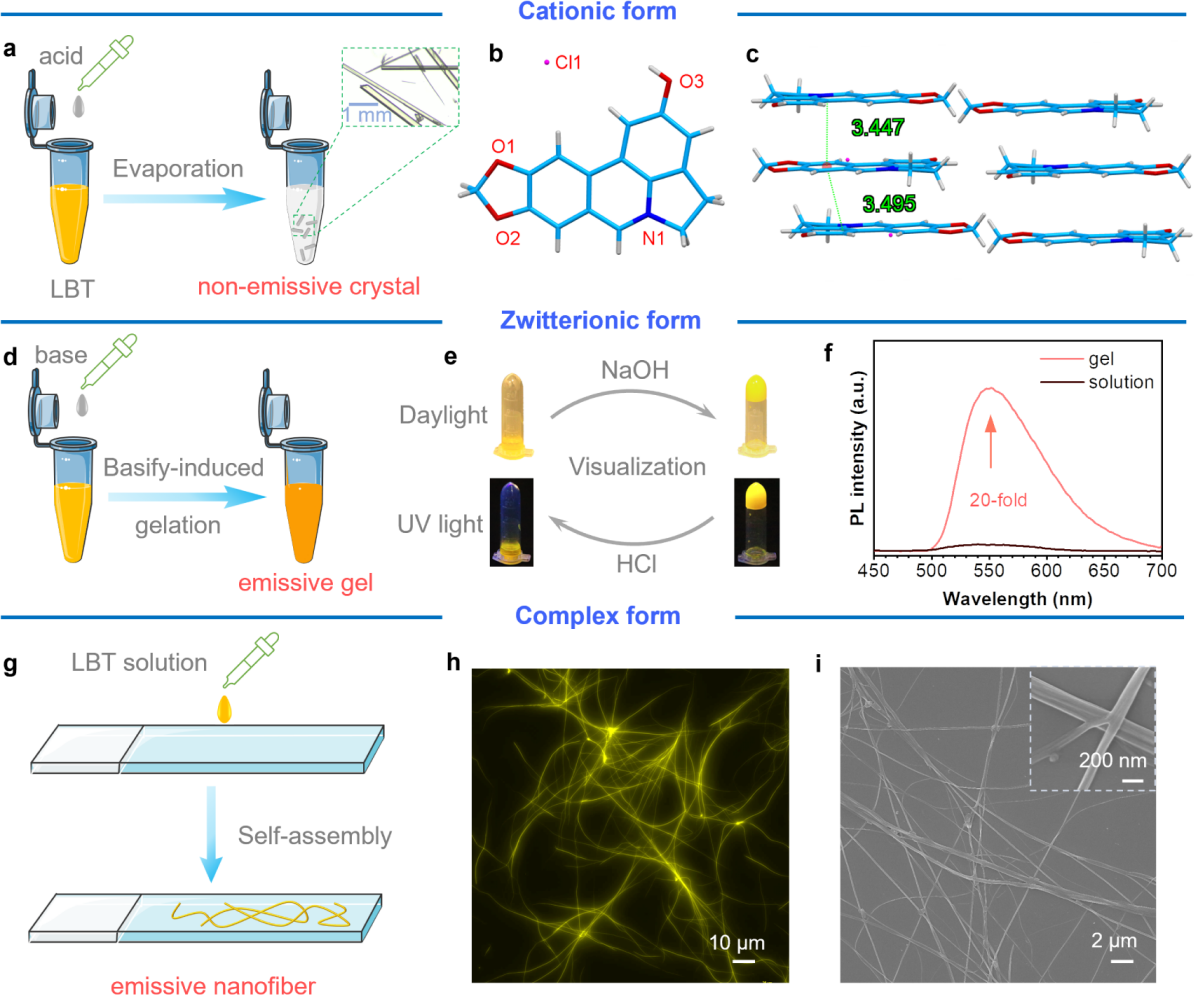
Photophysical properties of LBT in different aggregate forms. (**a**) Illustration of crystal growth of cationic LBT with its (**b**) single crystal structure and (**c**) packing mode. (**d**) Illustration of the gelation process of zwitterionic LBT with its (**e**) corresponding images and (**f)** PL spectra (λ_ex_ = 420 nm) before and after gelation. (**g**) A schematic diagram showing the self-assembly process of LBT and its (**h**) fluorescence (λ_ex_ = 450–490 nm; emission was collected using a long-pass filter with a cutoff wavelength of 515 nm) and (**i**) SEM images.

Aggregates often display distinct characteristics compared with their molecular counterparts, making them a subject of significant interest for further structure–property investigations. In addition to studying LBT at the molecular level, we also investigated the properties of LBT in its aggregate state. As illustrated in Figure 2a, the needlelike single crystals of LBT in their cationic form suitable for single-crystal X-ray diffraction (SXRD) analyses were obtained by slow solvent evaporation of acidified LBT solutions (Table S1). The SXRD results revealed that the LBT molecule adopted an almost planar conformation (Figure 2b). Additionally, stacked structures were observed, characterized by strong intermolecular π···π interactions with distances below 3.5 Å. These interactions likely contribute to the nonemissive nature of LBT in its crystalline state (Figure 2c). In contrast, the alkalization of saturated LBT in aqueous solutions induced gelation, as illustrated in Figure 2d. Upon the addition of NaOH, the solution rapidly transformed into a gel state. In addition, the gel-to-solution transitions can be induced by the addition of HCl. The LBT hydrogel showed reversible responsiveness to varied pH values (Figure 2e). Its emission intensity showed an over 20-fold increase after gelation (Figure 2f). Interestingly, when the water was evaporated, the self-assembly of LBT into nanofibers exhibiting a yellow emission was observed under a fluorescence microscope (Figures 2g and S5). The imaging of LBT nanofibers using scanning electron microscopy (SEM) revealed that the nanofibers possessed a diameter below 100 nm, which was consistent with the observations made using a fluorescence microscope (Figure 2h,i). To gain a better understanding of the assembly process, positive electrospray ionization mass spectrometry (ESI-MS)^56^ was employed to elucidate the components of LBT fibers. Monomers (*m*/*z* 266.3), dimers (*m*/*z* 531.3), and trimers (*m*/*z* 796.4) of LBT were obtained (Figure S6), which indicates the favorable formation of cationic and zwitterionic complexes.

### Intracellular Nanofilaments Formation

We then explored the application of LBT in live-cell imaging. Before the cell imaging experiment, we used the Cell Counting Kit-8 (CCK-8) assay to assess the cytotoxicity of LBT in live cells.^57,58^ As illustrated in Figure S7, after incubation with 10 μM LBT for 24 h, the cell survival rates of HeLa and NIH/3T3 cells were about 70%. Live-cell fluorescence imaging of HeLa cells at 37 °C showed that only weak staining of cells was observed after 2 h of incubation with LBT (Figure S8). However, sporadic filaments were observed in some of the HeLa cells after 5 h of incubation. Similar findings were observed in NIH/3T3 cells, as illustrated in Figure S9.

**Figure 3:**
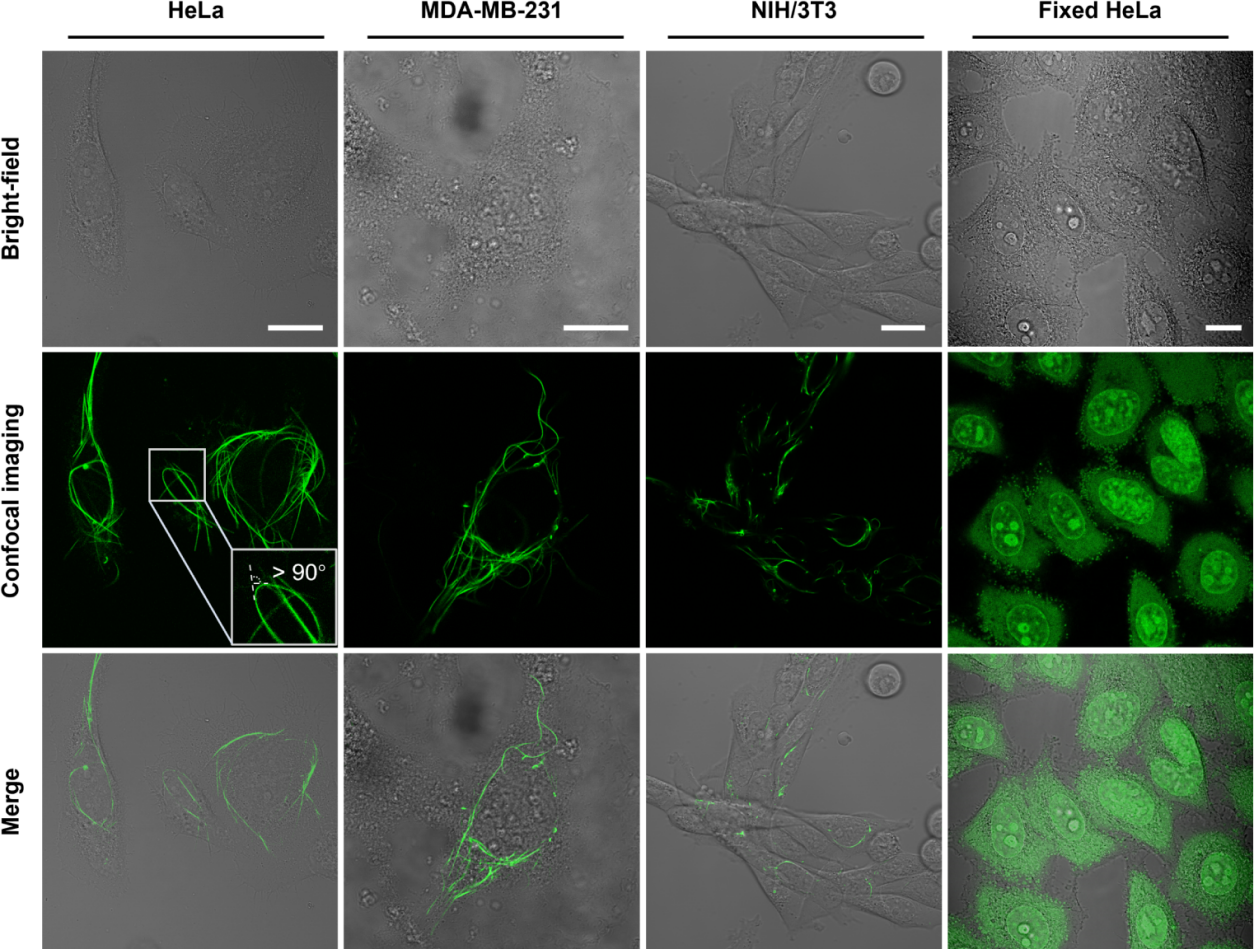
Representative bright-field and fluorescence images of live HeLa, MDA-MB-231, NIH/3T3 cells, and fixed HeLa cells incubated with LBT (10 μM). Cells were stimulated by a cold treatment during incubation (λ_ex_ = 488 nm, λ_em_ = 500–700 nm). Scale bars: 20 μm.

In our experiments, we unexpectedly found that the cold environment greatly facilitates the formation of LBT nanofibers inside the cells. While HeLa cells stained with LBT at 37 °C did not show obvious nanofiber formation, a cold treatment (5 min, 15 °C) of the LBT-stained cells could significantly induce LBT fiber assembly. After cold treatment and a subsequent 1-h incubation, robust intracellular LBT networks were observed (Figure S10), and the same method yielded similar results in the other cell lines (NIH/3T3, and MDA-MB-231) tested (Figure 3). In particular, the formation of LBT filament networks in the cellular environment encompasses three stages: (i) enrichment of LBT molecules with adequate concentrations; (ii) induction of intracellular LBT filament formation via a temporary 5 min cold treatment; and (iii) extension of LBT filaments in the cellular environment upon further incubation. Intracellular LBT nanofibers were completely confined inside the cells with some fibers over 100 μm long or even with a bending angle greater than 90°. The LBT fibers seem to be physically harmless to the cells, as the cells with more fibers (with short cold treatment) demonstrated higher cell viability than the cells with fewer fibers (without short cold treatment; Figure S11).

### LBT Nanofibers Physically Associate with Mitochondria

**Figure 4:**
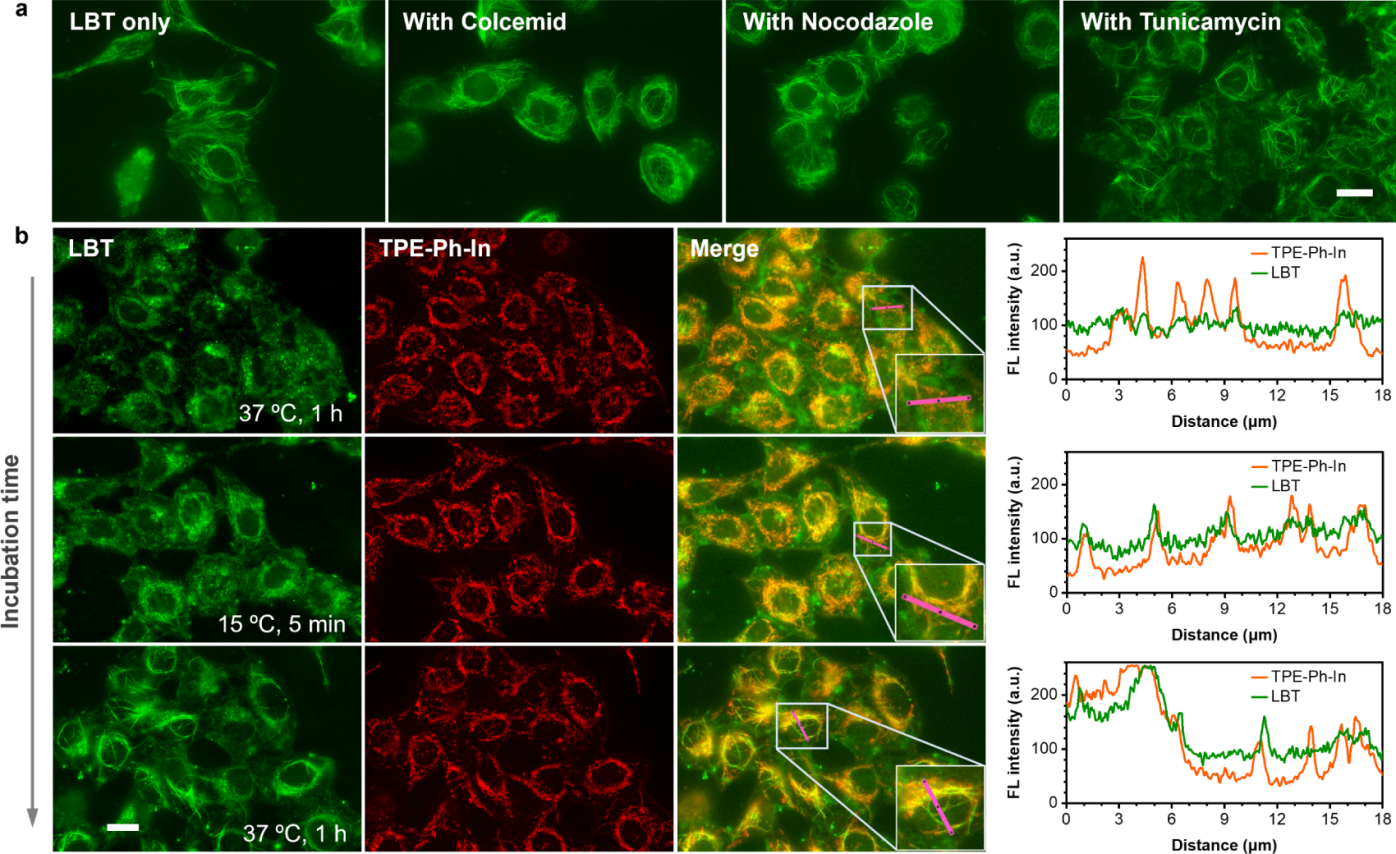
(**a**) Fluorescence images of live LBT-stained HeLa cells pretreated with colcemid, nocodazole, or cotreated with tunicamycin. All cells were imaged after the cold treatment. Scale bars: 20 μm. (**b**) Line scan analysis of LBT and TPE-Ph-In in live HeLa cells with the corresponding fluorescence (FL) intensity along the solid pink line. Cells at the three indicated time points were imaged. Green fluorescence represents LBT (λ_ex_ = 465–495 nm, λ_em_ = 512–558 nm); red fluorescence represents TPE-Ph-In (λ_ex_ = 509–519 nm; emission was collected using a long-pass filter with a cutoff wavelength of 590 nm). Scale bars: 20 μm.

To further investigate the mechanism of intracellular LBT assembly, the relationship between LBT nanofibers and endogenous tubule-shaped networks, including microtubules and the endoplasmic reticulum (ER), was examined. The inhibition of tubulin polymerization using two microtubule destabilizers,^59^ colcemid and nocodazole, did not affect the formation of LBT nanofibers in HeLa cells (Figure 4a). Similarly, treatment with tunicamycin, which induces ER stress,^60^ did not significantly impact the formation of LBT nanofibers. These observations suggest that LBT fibers can form independently of the networks of microtubules or the ER. We proceeded to investigate whether LBT nanofiber formation is correlated to the tubular network of mitochondria. To explore the relationship, we costained the cells with LBT and TPE-Ph-In,^61−63^ a mitochondria-specific probe. We confirmed that there is no fluorescence crosstalk between TPE-Ph-In and LBT (Figure S12). The resulting images revealed no clear spatial relationship between the signal of LBT and TPE-Ph-In (Figure 4b). Line scan analysis also supported such funding. However, after subjecting the cells to cold treatment, we observed that the LBT signal overlaid with TPE-Ph-In signals in mitochondria. Following a 1 h incubation period, LBT fiber networks were formed in HeLa cells, resulting in less overlapping with mitochondria. These observations were consistent with the results obtained using another mitochondrial probe, tetramethylrhodamine methyl ester (TMRM) (Figure S13).

### LBT-Nanofiber-Mediated Synchronization of ΔΨ_m_ Oscillations

**Figure 5:**
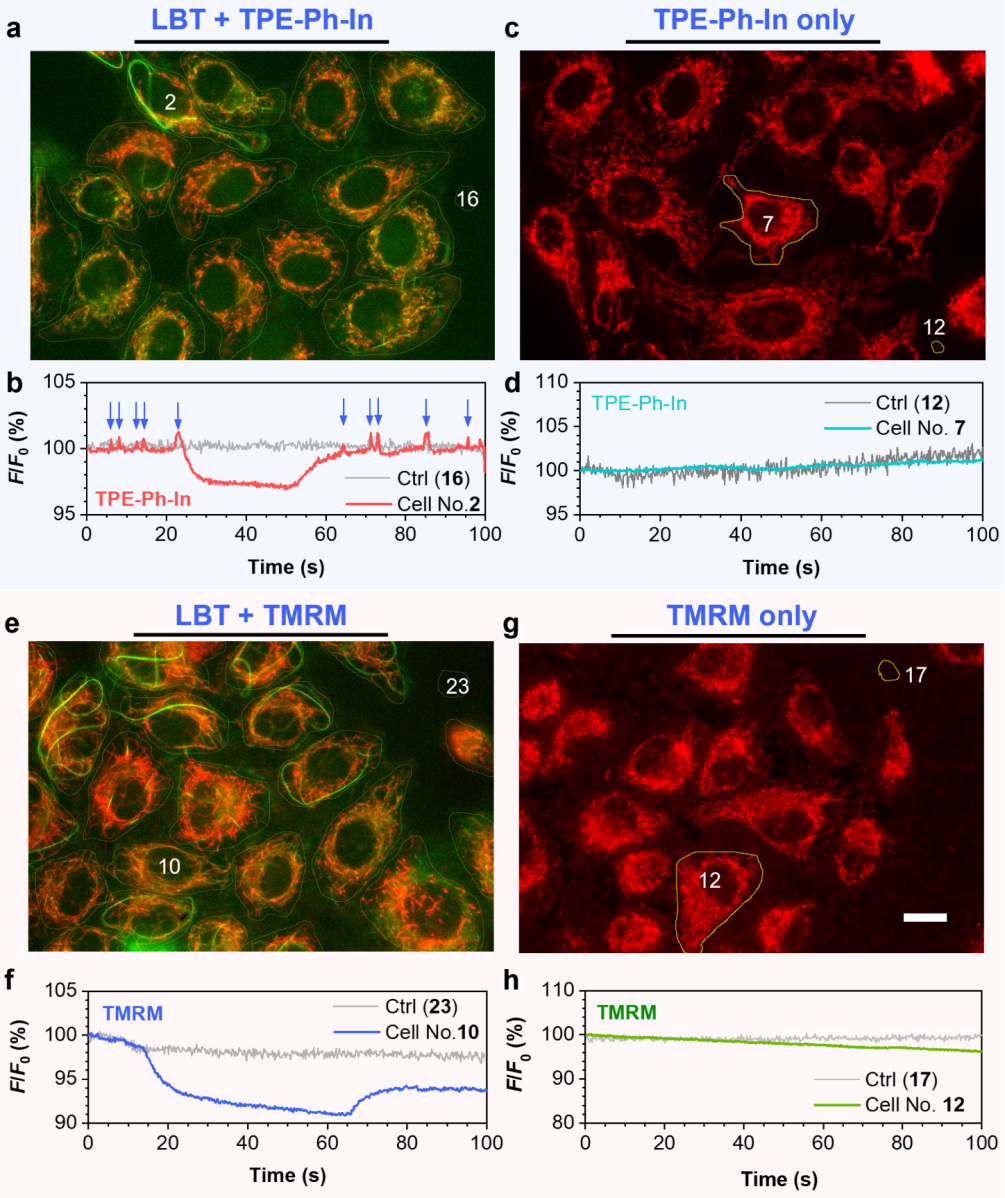
(**a**) Fluorescence image of living HeLa cells. Cells were incubated with LBT (10 μM) and TPE-Ph-In (5 μM) for 2 h followed by the cold treatment. (**b**) Time course analysis of TPE-Ph-In fluorescence intensity of cell No. 2 in (**a**), where *F* and *F*_0_ represent the real-time and initial fluorescence intensities, respectively. (**c**) The fluorescence image of living HeLa cells. Cells were incubated with TPE-Ph-In (5 μM) only for 2 h followed by the cold treatment. (**d**) Time course analysis of TPE-Ph-In fluorescence intensity of cell No. 7 in (**c**). (**e**) The fluorescence image of living HeLa cells. Cells were incubated with LBT (10 μM) and TMRM (20 nM) for 2 h, followed by cold treatment. TMRM was used in nonquenching mode. (**f**) Time course analysis of TMRM fluorescence intensity of cell No. 10 in (**e**). (**g**) The fluorescence image of HeLa cells. Cells were incubated with TMRM (20 nM) only for 2 h, followed by the cold treatment. TMRM was used in nonquenching mode. (**h**) Time course analysis of TMRM fluorescence intensity of cell No. 12 in (**g**). Scale bar: 20 μm. Green fluorescence represents LBT (λ_ex_ = 465–495 nm, λ_em_ = 512–558 nm); red fluorescence represents TPE-Ph-In (**a** and **c**) or TMRM (**e** and **g**) (λ_ex_ = 509–519 nm; emission was collected using a long-pass filter with a cutoff wavelength of 590 nm).

During fluorescence imaging of HeLa cells stained with LBT and TPE-Ph-In, it was observed that the TPE-Ph-In channel exhibited spontaneous flashings at a cellular level, which indicated fluctuation in mitochondrial membrane potential.^4,61^ To further investigate the flashing signal of ΔΨ_m_, specific cells were selected as a region of interest (ROI) and the red fluorescence intensity was recorded over a period of 100 s. The blank region (No. 16) represented the background fluorescence intensity. Interestingly, two types of ΔΨ_m_ oscillation patterns were observed (Figures 5a,b and S14). The first type consisted of transient spikes in fluorescence intensity, with a full width at half-maximum (fwhm) of approximately 0.5 s (*n* = 211 from three independent experiments), indicated by the blue arrows. The second type comprised oscillations lasting for more than half a minute. In contrast, cells stained only with TPE-Ph-In did not exhibit any noticeable signal fluctuation (Figures 5c,d and S15). TPE-Ph-In was reported to be sensitive to ΔΨ_m_ fluctuation, and we then hypothesized that the spikes and oscillations in fluorescence intensity were caused by changes in ΔΨ_m_. To further verify the changes of ΔΨ_m_, a well-established commercial fluorescent probe for ΔΨ_m_, TMRM was used. The fluorescence intensity fluctuations of HeLa cells stained with LBT and TMRM were then recorded. Only longer oscillations were observed, with the entire event lasting approximately 60 s (*n* = 19 from three independent experiments). The TMRM signal displayed a decrease in fluorescence intensity within about 1.6 s, followed by a plateau and a slow recovery back to the baseline level, representing synchronized depolarization and repolarization processes (Figures 5e,f and S16). The time course oscillations of the traditional ΔΨ_m_ probe TMRM were similar to the reported phenomena of mitochondrial metabolism oscillation.^64,65^ However, no fluorescence signal fluctuation was observed in cells stained only with TMRM (Figures 5g,h and S17). Unlike TPE-Ph-In, the transient spike signals were not recorded by TMRM, which suggested a low temporal sensitivity of TMRM.

**Figure 6:**
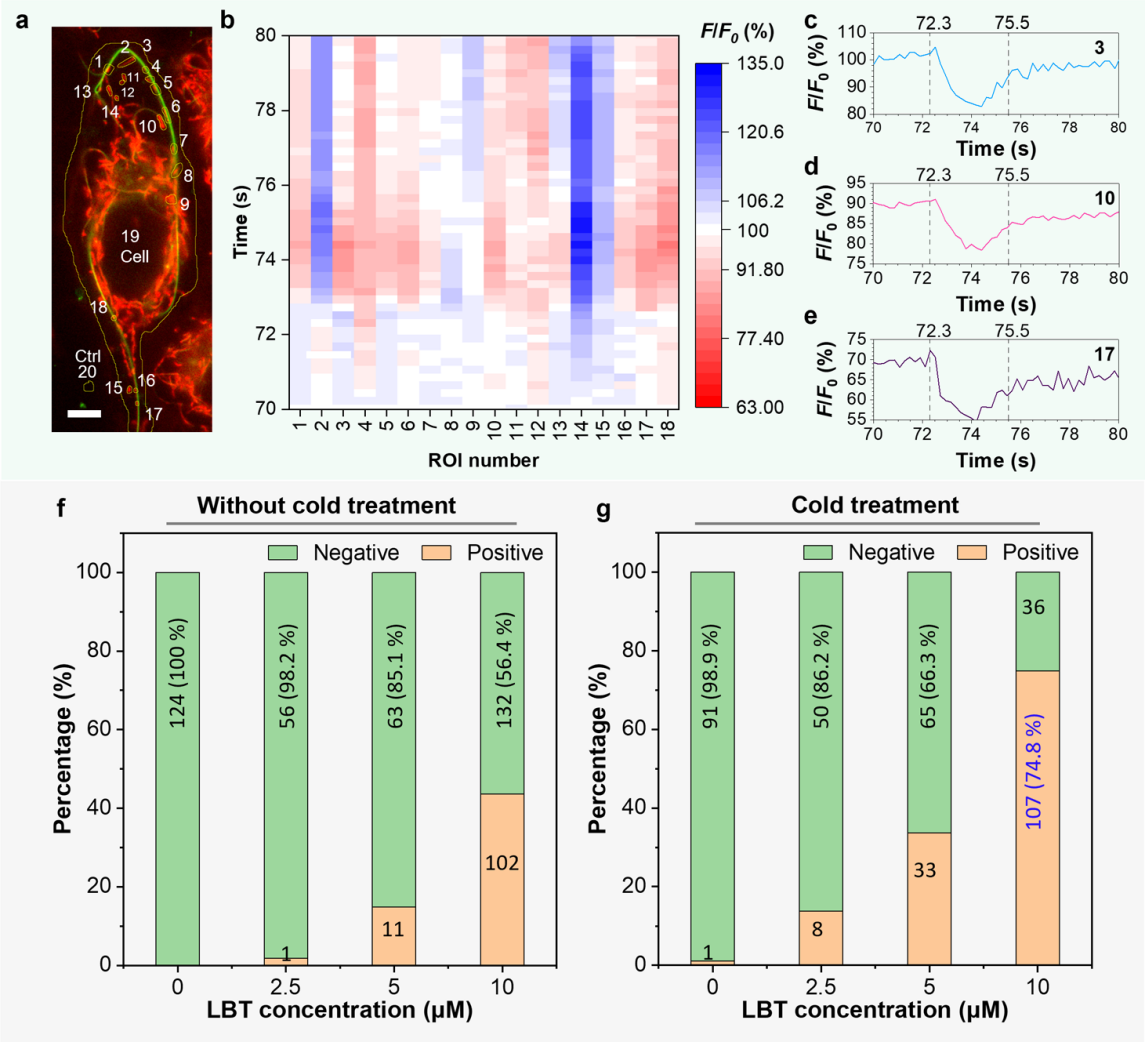
(**a**) Fluorescence images of living HeLa cells. Cells were incubated with LBT (10 μM) and TPE-Ph-In (5 μM) for 2 h, followed by cold treatment. Scale bar: 5 μm. (**b–e**) Time course analysis of TPE-Ph-In fluorescence intensity of individual mitochondria in (**a**). Mitochondria were randomly picked and labeled as a fluorescence region of interest (ROI) and their fluorescence intensity. *F* and *F*_0_ represent the real-time and initial fluorescence intensities, respectively. (**f–g**) Percentage of cells with ΔΨ_m_ signal oscillations incubated with TPE-Ph-In (5 μM) and different concentrations of LBT with or without cold treatment.

To evaluate the frequency and amplitude of ΔΨ_m_ signal flashing induced by LBT fibers, we analyzed the spatial-temporal relationship of the flashing events in individual mitochondria of the same cell. The cell shown in Figure 6a, selected from the representative fluorescence image in Figure S18, is given as an example. In this cell, distant mitochondria 3, 10, and 17, which were connected to the LBT fibers, exhibited in-phase ΔΨ_m_ signal flashing (Figure 6b–e). In comparison, other mitochondria that were not connected to the LBT networks, such as mitochondria number 2, 14, and 15, displayed uncoupled ΔΨ_m_ signal flashing. Subsequently, individual mitochondria within the same cell were selected as ROIs. Over 50% of the randomly selected mitochondria demonstrated synchronized depolarization and repolarization during the recording period (Figure S19). These results suggest that synchronized ΔΨ_m_ oscillations in these LBT-nanofiber-presented cells are observed.

The statistical study of flashing events was also conducted, as shown in Figure 6f,g. With an increase in LBT concentration from 0 to 10 μM, there was a corresponding rise in the percentage of cells exhibiting fluorescence flashing. After additional cold treatment, 74.8% of the 10 μM LBT stained cells showed flashing events. Since the cold treatment greatly facilitates intracellular LBT nanofiber formation, these fibers appear to contribute to the ΔΨ_m_ oscillations. To further investigate the nature of these fibers, the electrical conductivity of the LBT nanofiber film was measured, showing a nonlinear behavior that was similar to semiconductors (Figure S20). This confirms that LBT fibers are capable of transmitting electronic signals.^66^ Based on these findings, it is postulated that the semiconductive LBT nanofibers establish physical connections between mitochondria, allowing for the conduction of electrical signals and the amplification of spontaneous spikes. Consequently, the flashing events were eventually detected *in situ*.

### K^+^ Relevant Mitochondrial Flashing

**Figure 7:**
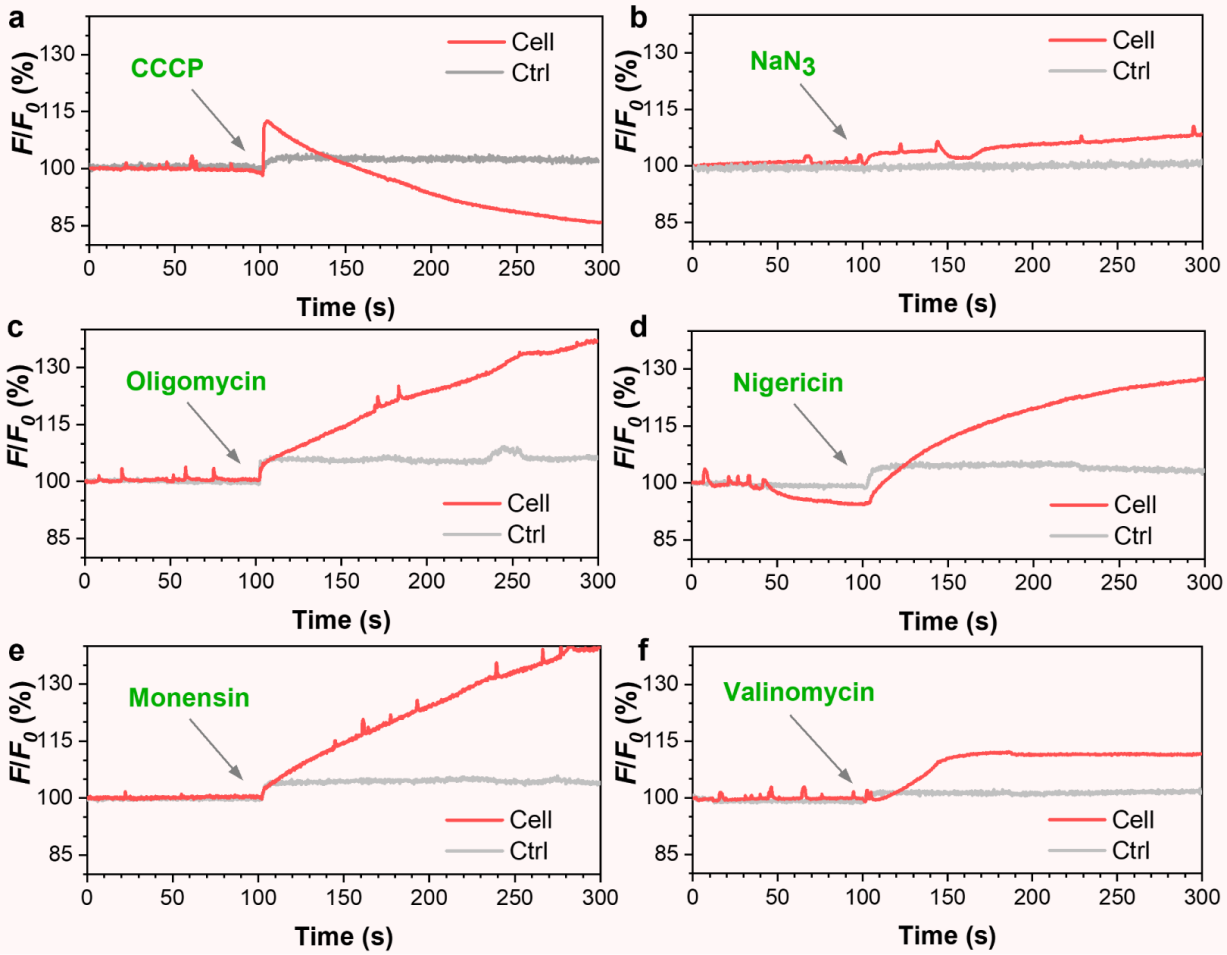
Relative fluorescence intensity changes of TPE-Ph-In in live HeLa cells stained with LBT and TPE-Ph-In before and after the treatment of CCCP (**a**), NaN_3_ (**b**), oligomycin (**c**), nigericin (**d**), monensin (**e**), and valinomycin (**f**), respectively. Arrows indicate the time points (100 s) of adding drugs. *F* and *F*_0_ represent the real-time and initial fluorescence intensities, respectively.

To further investigate the physiological relevance to the synchronous oscillation of ΔΨ_m_, we treated the entire cell as the region of interest (ROI) and studied the influencing factors by observing the fluorescence changes in the cell before and after pharmacological treatments. We first treated the TPE-Ph-In-stained LBT-nanofiber-presented cells with carbonyl cyanide *m*-chlorophenylhydrazone (CCCP), a proton-specific ionophore that dissipates ΔΨ_m_ across the mitochondrial inner membrane. The TPE-Ph-In signals substantially increased shortly after CCCP was added to the medium. This increase was followed by a continuous decline in the TPE-Ph-In signals without any spontaneous spikes, confirming the dependence of the flash on the ΔΨ_m_ value (Figure 7a). Treatment with sodium azide (NaN_3_), which is a potent inhibitor of cellular respiration, particularly of the electron transport chain in mitochondria, did not affect the flashing of the TPE-Ph-In signals (Figure 7b). When oligomycin, a mitochondrial ATPase inhibitor used to increase ΔΨ_m_, was introduced into the medium, the TPE-Ph-In signals across the entire field of view significantly elevated, while the spontaneous spikes persisted. This indicated that although LBT-nanofiber-induced TPE-Ph-In flashing relies on ΔΨ_m_ it is unrelated to the transient change of proton gradient across the inner mitochondrial membrane (Figure 7c). Furthermore, the addition of nigericin, an ionophore that facilitates selective exchange of K^+^ and H^+^, resulted in the abolition of TPE-Ph-In signal spikes (Figure 7d). This observation suggests that ΔΨ_m_ oscillation is influenced by the concentration gradient of these two ions across the inner mitochondrial membrane. In contrast, treatment with monensin, which promotes Na^+^/H^+^ exchange and decreases the mitochondrial matrix pH, did not affect the occurrence of spontaneous spikes (Figure 7e). Finally, the addition of valinomycin, commonly used to eliminate the transmembrane potential by collapsing the K^+^ concentration gradient, completely abolished the ΔΨ_m_ oscillation as indicated by the TPE-Ph-In signal (Figure 7f). All control trials were included in Figure S21, which describes the intensity changes in TPE-Ph-In fluorescence. These results demonstrated that there was no fluorescence oscillation before or after the drug was added when living HeLa cells were treated solely with TPE-Ph-In. This further confirmed the crucial role of LBT nanofibers in the synchronized ΔΨ_m_ oscillation. The frequency of ΔΨ_m_ fluctuations in HeLa cells before and after treatment with specific drugs was statistically analyzed (Figure S22). The data represent the average of at least three independent experiments, demonstrating solid evidence of K^+^-dependent mitochondrial flashing. Valinomycin treatment has yielded similar results in the Huh-7 cell line (Figure S23), corroborating that it is a common phenomenon.

Collectively, our findings reveal the existence of a fast, frequent, low amplitude, and K^+^ concentration gradient-dependent ΔΨ_m_ fluctuation, which is distinct from previously reported mitochondrial permeability transition pore (mPTP)-dependent ΔΨ_m_ oscillations. Based on these observations, we speculate that the rapid opening and closing of voltage-gated K^+^ ion channels may be the primary cause of this ΔΨ_m_ signal flashing. Although the mitochondrial-located voltage-gated potassium channel Kv1.3 has been associated with abnormal mitochondrial function, apoptosis, and abnormal steady ΔΨ_m_ through function inhibition or gene silencing,^67,68^ the regulation of ΔΨ_m_ through this channel has not been investigated, not to mention the mechanism of the regulation. Our study provides an easy but powerful tool to observe transient mitochondrial membrane potential oscillation, which could explore the emerging field of regulation and maintenance of transient mitochondrial membrane potential homeostasis.

## Conclusion

To summarize, a comprehensive investigation was conducted on the photophysical properties of a small-molecule LBT, encompassing its fluorescence property and emission mechanism at the molecular and aggregate levels. LBT could form crystals, gels, or nanofiber filaments in aqueous solutions with different pH values. Intracellularly, LBT was found to develop filament networks in live cells that were induced at low temperatures with proximity to mitochondria. Given the localization and electrical conductivity in mitochondria, the LBT filament network is capable of operating as an intracellular “nano-cable”, enabling synchronization and amplification of ΔΨ_m_ oscillations. As a result, metabolic oscillation of ΔΨ_m_ or mitochondrial K^+^ flux with spontaneous ΔΨ_m_ flashing at a cellular level can be fluorescently observed in real-time through the ΔΨ_m_-sensitive probe TMRM or TPE-Ph-In. This work not only demonstrates the possibility of manipulating the connectivity of mitochondria but also introduces a platform facilitating the analysis of mitochondria within individual cells, highlighting synchronized cellular activities and enhanced ΔΨ_m_ signal detection.

## Methods and Materials

### Materials and General Instruments

All chemicals and reagents were commercially available and used as received without further purification. LBT was purchased from Psaitong (China) and used without further purification. The purity has been confirmed by ^1^H NMR, high-performance liquid chromatography (HPLC), and high-resolution mass spectrometry (HRMS) analysis (Figures S24–S26). CCK-8 kits were purchased from Beyotime. Dulbecco’s modified Eagle’s medium (DMEM), Dulbecco’s phosphate buffered saline (DPBS), fetal bovine serum (FBS), trypsin-EDTA, and penicillin-streptomycin were obtained from Gibco. Tunicamycin, colcemid, nocodazole, and propidium iodide (PI) were purchased from Thermo Fisher Scientific. Milli-Q water was supplied by a Milli-Q Plus System (Millipore Corporation, United States). All of the solvents for optical spectroscopic studies were of spectroscopic grade. UV–vis absorption spectra were performed using a PerkinElmer Lambda 365 spectrophotometer. Fluorescence spectra were obtained by using a Horiba Duetta spectrofluorometer with a 10 mm quartz cuvette. Fluorescence images were collected on an Olympus IX71 inverted fluorescence microscope or Zeiss LSM 880 confocal laser scanning microscope. Scanning electron microscopy (SEM) micrographs were collected on a field emission scanning electron microscope (model: JSM-6700F). ChatGPT was utilized to assist in correcting grammar mistakes and providing language suggestions for this manuscript.

### Single Crystal Cultivation

The single crystals of LBT in its cationic form were grown using the slow evaporation method at room temperature. An initial stock solution of LBT was prepared by dissolving the compound in a minimal volume of acetonitrile to achieve a supersaturated state. This solution was then filtered by using a 0.22 μm syringe filter to remove any particulate matter. A small volume of the HCl solution was carefully added. The formed needlelike crystals were examined for quality and size under a polarizing microscope before being selected for X-ray diffraction analysis.

### Computational Details

LBT in its cationic form and zwitterionic form were fully optimized with the density functional theory (DFT) method by using the M06-2X density functional and 6-31+G(d,p) basis set. Analytical frequency calculations were also performed at the same level of theory to confirm that the optimized structures were at a minimum point. The frontier molecular orbitals (FMO) were displayed by using the IQmol molecular viewer package. All of the quantum chemical calculations were carried out using the Gaussian 09 program.

### Cell Culture

HeLa cells, NIH/3T3 cells, MDA-MB-231, and Huh-7 cells were cultured in DMEM containing 10% FBS in a 5% CO_2_ humidified incubator at 37 °C. Once the cells reached 80–90% confluence, they were dissociated into single cells with 0.05% trypsin-EDTA at 37 °C for 5 min and passaged at a ratio of 1:4–1:10 in one cell culture dish.

### Cell Fixation

HeLa cells were fixed with 4% PFA for 10–20 min at room temperature. Fixed cells were then washed with DPBS three times to remove the fixative solution.

### CCK-8 Assay for the Determination of Cell Cytotoxicity

The cytotoxicity on cells was determined by the standard WST-8 (2-(2-methoxy-4-nitrophenyl)-3-(4-nitrophenyl)-5-(2,4-disulfophenyl)-2H-tetrazolium, monosodium salt) (CCK-8) assay. HeLa cells and NIH/3T3 cells were seeded at a density of 7 × 10^3^ cells and 1.4 × 10^4^ cells per well, respectively, in standard 96-well clear microplates with 100 μL of culture medium and cultured overnight to reach 70–80% confluence. After that, the medium was replaced with 100 μL of fresh medium containing different concentrations of LBT (0, 2.5, 5, 10, 20, 30, and 40 μM), and water was used as vehicle control. After 24 h of incubation, 10 μL of 12 mM CCK-8 stock solution mixed with 90 μL of the abovementioned LBT containing fresh medium was added to each well for an additional 1 h of incubation. For cold treatment, cells were stained with 10 μM LBT for 1 h in the incubator followed by 5 min cold treatment (15 °C) and then another 1 h in the incubator. Control cells without LBT staining were incubated for 2 h in the incubator. After incubation, cells were changed to CCK-8 containing fresh medium and incubated for an additional 1 h in the incubator. The absorbance at 450 nm (OD_450_) was measured using the SpectraMax M2 microplate reader (Molecular Devices). Cell viability (%) was calculated: (OD_450_ sample/OD_450_ control) × 100%.

### Fluorescence Imaging of LBT

All fluorescence imaging was conducted by a fluorescence microscope or confocal laser scanning microscope. DMEM without phenol red was used as a staining and imaging medium. Cells were sequentially incubated with 10 μM LBT at 37 °C for 1 h, 15 °C for 5 min, and 37 °C for another 1 h. After the incubation, the cells were directly imaged without washing to reduce the perturbation of the LBT filaments. Confocal imaging was performed using the Zeiss LSM 880 confocal laser scanning microscope equipped with a Plan-Apochromat 63 × /1.4 NA oil objective lens, a photomultiplier tube, and a Gallium arsenide phosphide detector driven by the ZEN software (Carl Zeiss). For confocal imaging of LBT, the 488 nm laser was used and the emission between 500 and 700 nm were detected. For fluorescent imaging of LBT with inverted microscope, λ_ex_ = 465–495 nm, λ_em_ = 512–558 nm except in Figure 2 (λ_ex_ = 450–490 nm, λ_em_ = LP 515 nm); for fluorescent imaging of TPE-Ph-In or TMRM with inverted microscope, λ_ex_ = 509–519 nm; emission was collected using a long-pass filter with a cutoff wavelength of 590 nm. All filters used for excitation and emission were as indicated in the figure captions. All videos of TPE-Ph-In and TMRM were taken at a speed of 5 frames per second. Digital images were captured and processed by ZEN software (ZEN 2.5 lite) in grayscale and pseudo color.

### Image Processing

Images were processed by Fiji (ImageJ 1.53s) software (NIH). Z-stacked images acquired on the Zeiss LSM confocal were processed on ImageJ. Images for colocalization studies were first processed with the background subtract function using Fiji and then analyzed with the colocalization function on Fiji.

### Statistics and Reproducibility

All microscopy experiments were repeated with similar results. The details are listed in Table S2 (Supporting Information).

## Supporting Information Available

Figure S1–S26: emission spectra of LBT; normalized excitation spectra of LBT; ^1^H NMR spectra of LBT treated with HCl or NaOH; HOMO and LUMO plots and corresponding energy gaps from DFT calculations; fluorescence imaging of self-assembly LBT nanofibers with different concentrations; ESI-MS calcd. for C16H12NO3^+^, C32H23N2O6^+^, and C48H34N3O9^+^; the CCK8 assay of LBT; cellular uptake and distribution of LBT in HeLa cells; cellular uptake and distribution of LBT in NIH/3T3 cells; LBT staining of live HeLa and NIH/3T3 cells; the CCK8 assay of LBT with the cold treatment; fluorescence imaging of LBT stained HeLa cells; colocalization of LBT and TMRM; fluorescence images of HeLa cells incubated with TPE-Ph-In and LBT with the cold treatment; fluorescence images of HeLa cells incubated with TPE-Ph-In; merged fluorescence images of HeLa cells incubated with TMRM and LBT with the cold treatment; fluorescence images of HeLa cells incubated with TMRM; fluorescence images of HeLa cells incubated with TPE-Ph-In and LBT with the cold treatment; fluorescence images of the HeLa cell No. 10 from Figure S16; the electrode device to measure the electrical conductivity of the LBT nanofiber; fluorescence changes of TPE-Ph-In-stained HeLa cells before and after different drug treatments; statistical analysis of ΔΨ_m_ fluctuation frequency of HeLa and Huh-7 cells; ^1^H NMR, HPLC and HRMS spectra of LBT. Table S1–S2: crystal data and structure refinement for the cationic form of LBT; statistics and reproducibility of cell images (PDF)

## Accession Codes

CCDC 2202095 contains the supplementary crystallographic data for this paper. These data can be obtained free of charge via www.ccdc.cam.ac.uk/data_request/cif, or by emailing data_request@ccdc.cam.ac.uk, or by contacting The Cambridge Crystallographic Data Centre, 12 Union Road, Cambridge CB2 1EZ, UK; fax: + 44 1223 336 033. All data used to support the findings in the paper and Supporting Information are available from the corresponding authors upon reasonable request.

## References

[ref1] Nunnari J.; Suomalainen A. Mitochondria: In Sickness and In Health. Cell 2012, 148, 1145–1159. 10.1016/j.cell.2012.02.035. 22424226 PMC5381524

[ref2] Zorova L. D.; Popkov V. A.; Plotnikov E. Y.; Silachev D. N.; Pevzner I. B.; Jankauskas S. S.; Babenko V. A.; Zorov S. D.; Balakireva A. V.; Juhaszova M.; et al. Mitochondrial Membrane Potential. Anal. Biochem. 2018, 552, 50–59. 10.1016/j.ab.2017.07.009. 28711444 PMC5792320

[ref3] O’Reilly C. M.; Fogarty K. E.; Drummond R. M.; Tuft R. A.; Walsh J. V. Quantitative Analysis of Spontaneous Mitochondrial Depolarizations. Biophys. J. 2003, 85, 3350–3357. 10.1016/S0006-3495(03)74754-7. 14581236 PMC1303612

[ref4] Perry S. W.; Norman J. P.; Barbieri J.; Brown E. B.; Gelbard H. A. Mitochondrial Membrane Potential Probes and the Proton Gradient: A Practical Usage Guide. BioTechniques 2011, 50, 98–115. 10.2144/000113610. 21486251 PMC3115691

[ref5] Kamo N.; Muratsugu M.; Hongoh R.; Kobatake Y. Membrane Potential of Mitochondria Measured with an Electrode Sensitive to Tetraphenyl Phosphonium and Relationship between Proton Electrochemical Potential and Phosphorylation Potential in Steady State. J. Membr. Biol. 1979, 49, 105–121. 10.1007/BF01868720. 490631

[ref6] Zorov D. B.; Juhaszova M.; Sollott S. J. Mitochondrial Reactive Oxygen Species (ROS) and ROS-Induced ROS Release. Physiol. Rev. 2014, 94, 909–950. 10.1152/physrev.00026.2013. 24987008 PMC4101632

[ref7] Liu S.; Liu S.; He B.; Li L.; Li L.; Wang J.; Cai T.; Chen S.; Jiang H. Oxphos Deficiency Activates Global Adaptation Pathways to Maintain Mitochondrial Membrane Potential. EMBO Rep. 2021, 22 (4), e51606 10.15252/embr.202051606. 33655635 PMC8025004

[ref8] Narendra D.; Tanaka A.; Suen D. F.; Youle R. J. Parkin Is Recruited Selectively to Impaired Mitochondria and Promotes Their Autophagy. J. Cell Biol. 2008, 183, 795–803. 10.1083/jcb.200809125. 19029340 PMC2592826

[ref9] Green D. R.; Reed J. C. Mitochondria and Apoptosis. Science 1998, 281, 1309–1312. 10.1126/science.281.5381.1309. 9721092

[ref10] Iijima T. Mitochondrial Membrane Potential and Ischemic Neuronal Death. Neurosci. Res. 2006, 55, 234–243. 10.1016/j.neures.2006.04.005. 16716421

[ref11] Sifaoui I.; Reyes-Batlle M.; López-Arencibia A.; Chiboub O.; Rodríguez-Martín J.; Rocha-Cabrera P.; Valladares B.; Piñero J. E.; Lorenzo-Morales J. Toxic Effects of Selected Proprietary Dry Eye Drops on Acanthamoeba. Sci. Rep. 2018, 8 (1), 8520 10.1038/s41598-018-26914-3. 29867132 PMC5986802

[ref12] Owen M.; Doran E.; Halestrap A. Regulation of Metabolism-Evidence That Metformin Exerts Its Anti-Diabetic Effects through Inhibition of Complex 1 of the Mitochondrial Respiratory Chain. Biochem. J. 2000, 348, 607–614. 10.1042/bj3480607. 10839993 PMC1221104

[ref13] Connolly N. M. C.; Theurey P.; Adam-Vizi V.; Bazan N. G.; Bernardi P.; Bolaños J. P.; Culmsee C.; Dawson V. L.; Deshmukh M.; Duchen M. R.; Düssmann H.; et al. Guidelines on Experimental Methods to Assess Mitochondrial Dysfunction in Cellular Models of Neurodegenerative Diseases. Cell Death Differ. 2018, 25 (3), 542–572. 10.1038/s41418-017-0020-4. 29229998 PMC5864235

[ref14] Fulda S.; Galluzzi L.; Kroemer G. Evasion of Cell Death Is a Hallmark of Human Cancers and a Major Cause of Treatment Failure. Nat. Rev. Drug Discovery 2010, 9, 447–464. 10.1038/nrd3137. 20467424

[ref15] Li X.; Zhao Y.; Yin J.; Lin W. Organic Fluorescent Probes for Detecting Mitochondrial Membrane Potential. Coord. Chem. Rev. 2020, 420, 213419 10.1016/j.ccr.2020.213419.

[ref16] Amchenkova A. A.; Bakeeva L. E.; Chentsov Y. S.; Skulachev V. P.; Zorov D. B. Coupling Membranes as Energy-Transmitting Cables. I. Filamentous Mitochondria in Fibroblasts and Mitochondrial Clusters in Cardiomyocytes. J. Cell Biol. 1988, 107, 481–495. 10.1083/jcb.107.2.481. 3417757 PMC2115217

[ref17] Scholkmann F. Long Range Physical Cell-to-Cell Signalling Via Mitochondria inside Membrane Nanotubes: A Hypothesis. Theor. Biol. Med. Model. 2016, 13, 16 10.1186/s12976-016-0042-5. 27267202 PMC4896004

[ref18] De Giorgi F.; Lartigue L.; Ichas F. Electrical Coupling and Plasticity of the Mitochondrial Network. Cell Calcium. 2000, 28, 365–370. 10.1054/ceca.2000.0177. 11115375

[ref19] Li Y.; Huang D.; Jia L.; Shangguan F.; Gong S.; Lan L.; Song Z.; Xu J.; Yan C.; Chen T.; Tan Y. LonP1 Links Mitochondria–ER Interaction to Regulate Heart Function. Research 2023, 6, 0175 10.34133/research.0175. 37333972 PMC10275618

[ref20] Mendes A. C.; Baran E. T.; Reis R. L.; Azevedo H. S. Self-Assembly in Nature: Using the Principles of Nature to Create Complex Nanobiomaterials. Wiley Interdiscip. Rev.: nanomed. Nanobiotechnol. 2013, 5, 582–612. 10.1002/wnan.1238. 23929805

[ref21] Wagner B.; Tharmann R.; Haase I.; Fischer M.; Bausch A. R. Cytoskeletal Polymer Networks: The Molecular Structure of Cross-Linkers Determines Macroscopic Properties. Proc. Natl. Acad. Sci. U. S. A. 2006, 103, 13974–13978. 10.1073/pnas.0510190103. 16963567 PMC1599898

[ref22] Farge E. Mechanotransduction in Development. Curr. Top Dev. Biol. 2011, 95, 243–265. 10.1016/B978-0-12-385065-2.00008-6. 21501754

[ref23] Chagri S.; Ng D. Y. W.; Weil T. Designing Bioresponsive Nanomaterials for Intracellular Self-Assembly. Nat. Rev. Chem. 2022, 6, 320–338. 10.1038/s41570-022-00373-x. 37117928 PMC8972907

[ref24] Versluis F.; van Esch J. H.; Eelkema R. Synthetic Self-Assembled Materials in Biological Environments. Adv. Mater. 2016, 28, 4576–4592. 10.1002/adma.201505025. 27042774

[ref25] Wang H.; Feng Z.; Xu B. Bioinspired Assembly of Small Molecules in Cell Milieu. Chem. Soc. Rev. 2017, 46, 2421–2436. 10.1039/C6CS00656F. 28357433 PMC5480217

[ref26] He H.; Wang J.; Wang H.; Zhou N.; Yang D.; Green D. R.; Xu B. Enzymatic Cleavage of Branched Peptides for Targeting Mitochondria. J. Am. Chem. Soc. 2018, 140, 1215–1218. 10.1021/jacs.7b11582. 29328651 PMC5842676

[ref27] He P.; Li X.; Fan J.; Fan Y.; Yang P.; Li B.; Cong Y.; Yang C.; Zhang K.; Wang Z.; et al. Live Cells Process Exogenous Peptide as Fibronectin Fibrillogenesis in Vivo. CCS Chem. 2020, 2, 539–554. 10.31635/ccschem.020.201900117.

[ref28] Song B.-L.; Zhang X.-H.; Qiao Z.-Y.; Wang H. Peptide-Based Aiegens: From Molecular Design, Stimuli Responsiveness to Biomedical Application. CCS Chem. 2022, 4, 437–455. 10.31635/ccschem.021.202101231.

[ref29] Lin K.; Ma Z.; Li J.; Tang M.; Lindstrom A.; Ramachandran M.; Zhu S.; Lin T. Y.; Zhang L.; Li Y. Single Small Molecule-Assembled Mitochondria Targeting Nanofibers for Enhanced Photodynamic Cancer Therapy in Vivo. Adv. Funct. Mater. 2021, 31 (10), 2008460 10.1002/adfm.202008460. 37441230 PMC10338027

[ref30] Shi J.; Xu B. Nanoscale Assemblies of Small Molecules Control the Fate of Cells. Nano Today 2015, 10, 615–630. 10.1016/j.nantod.2015.09.001. 26900396 PMC4758372

[ref31] Geng J.; Li W.; Zhang Y.; Thottappillil N.; Clavadetscher J.; Lilienkampf A.; Bradley M. Radical Polymerization inside Living Cells. Nat. Chem. 2019, 11, 578–586. 10.1038/s41557-019-0240-y. 30988414

[ref32] Hu R.; Chen X.; Zhou T.; Si H.; He B.; Kwok R. T. K.; Qin A.; Tang B. Z. Lab-in-Cell Based on Spontaneous Amino-Yne Click Polymerization. Sci. China: Chem. 2019, 62, 1198–1203. 10.1007/s11426-019-9517-9.

[ref33] Cao H.; Yang Y.; Li J. AIEgen–Lipid Structures: Assembly and Biological Applications. Aggregate 2020, 1, 69–79. 10.1002/agt2.5.

[ref34] Cao S.; Shao J.; Abdelmohsen L. K. E. A.; Van Hest J. C. M. Amphiphilic AIEgen-Polymer Aggregates: Design, Self-Assembly and Biomedical Applications. Aggregate 2022, 3 (1), e128 10.1002/agt2.128.

[ref35] Hai Z.; Liang G. Intracellular Self-Assembly of Nanoprobes for Molecular Imaging. Adv. Biosyst. 2018, 2 (8), 1800108 10.1002/adbi.201800108.

[ref36] Li K.; Lyu Y.; Huang Y.; Xu S.; Liu H. W.; Chen L.; Ren T. B.; Xiong M.; Huan S.; Yuan L.; Zhang X. B.; Tan W. A De Novo Strategy to Develop NIR Precipitating Fluorochrome for Long-Term in Situ Cell Membrane Bioimaging. Proc. Natl. Acad. Sci. U. S. A. 2021, 118 (8), e2018033118 10.1073/pnas.2018033118. 33602816 PMC7923636

[ref37] Tan W.; Zhang Q.; Quinones-Frias M. C.; Hsu A. Y.; Zhang Y.; Rodal A.; Hong P.; Luo H. R.; Xu B. Enzyme-Responsive Peptide Thioesters for Targeting Golgi Apparatus. J. Am. Chem. Soc. 2022, 144, 6709–6713. 10.1021/jacs.2c02238. 35404599 PMC9069992

[ref38] Angerani S.; Lindberg E.; Klena N.; Bleck C. K. E.; Aumeier C.; Winssinger N. Kinesin-1 Activity Recorded in Living Cells with a Precipitating Dye. Nat. Commun. 2021, 12 (1), 1463 10.1038/s41467-021-21626-1. 33674590 PMC7935933

[ref39] Jeena M. T.; Palanikumar L.; Go E. M.; Kim I.; Kang M. G.; Lee S.; Park S.; Choi H.; Kim C.; Jin S.-M.; Bae S. C.; Rhee H. W.; Lee E.; Kwak S. K.; Ryu J. H. Mitochondria Localization Induced Self-Assembly of Peptide Amphiphiles for Cellular Dysfunction. Nat. Commun. 2017, 8, 26 10.1038/s41467-017-00047-z. 28638095 PMC5479829

[ref40] Zheng Z.; Chen P.; Xie M.; Wu C.; Luo Y.; Wang W.; Jiang J.; Liang G. Cell Environment-Differentiated Self-Assembly of Nanofibers. J. Am. Chem. Soc. 2016, 138, 11128–11131. 10.1021/jacs.6b06903. 27532322

[ref41] Zhou Z.; Maxeiner K.; Moscariello P.; Xiang S.; Wu Y.; Ren Y.; Whitfield C. J.; Xu L.; Kaltbeitzel A.; Han S.; et al. In Situ Assembly of Platinum(II)-Metallopeptide Nanostructures Disrupts Energy Homeostasis and Cellular Metabolism. J. Am. Chem. Soc. 2022, 144, 12219–12228. 10.1021/jacs.2c03215. 35729777 PMC9284552

[ref42] Weng Z.; Wang Z.; Zhang S.; Lv F.; Yang J.; Xu B. Chin. Sci. Bull. 1976, 6, 285–287.

[ref43] Casu L.; Cottiglia F.; Leonti M.; De Logu A.; Agus E.; Tse-Dinh Y. C.; Lombardo V.; Sissi C. Ungeremine Effectively Targets Mammalian as Well as Bacterial Type I and Type II Topoisomerases. Bioorg. Med. Chem. Lett. 2011, 21, 7041–7044. 10.1016/j.bmcl.2011.09.097. 22014547 PMC4551395

[ref44] Mbaveng A. T.; Bitchagno G. T. M.; Kuete V.; Tane P.; Efferth T. Cytotoxicity of Ungeremine Towards Multi-Factorial Drug Resistant Cancer Cells and Induction of Apoptosis, Ferroptosis, Necroptosis and Autophagy. Phytomedicine 2019, 60, 152832 10.1016/j.phymed.2019.152832. 31031043

[ref45] Gao Y.; Hu J.; Ju Y. Supramolecular Self-Assembly Based on Natural Small Molecules. Acta Chim. Sin. 2016, 74, 312–329. 10.6023/A16010016.

[ref46] Gu Y.; Zhao Z.; Su H.; Zhang P.; Liu J.; Niu G.; Li S.; Wang Z.; Kwok R. T. K.; Ni X. L.; et al. Exploration of Biocompatible AIEgens from Natural Resources. Chem. Sci. 2018, 9, 6497–6502. 10.1039/C8SC01635F. 30310579 PMC6115644

[ref47] Lee M. M. S.; Wu Q.; Chau J. H. C.; Xu W.; Yu E. Y.; Kwok R. T. K.; Lam J. W. Y.; Wang D.; Tang B. Z. Leveraging Bacterial Survival Mechanism for Targeting and Photodynamic Inactivation of Bacterial Biofilms with Red Natural AIEgen. Cell Rep. Phys. Sci. 2022, 3, 100803 10.1016/j.xcrp.2022.100803.

[ref48] Liu D.; Zhao Z.; Tang B. Z. Natural Products with Aggregation-Induced Emission Properties: From Discovery to Their Multifunctional Applications. Sci. Sin. Chim. 2022, 52, 1524–1546. 10.1360/SSC-2022-0082.

[ref49] Yuan H.; Jiang A.; Fang H.; Chen Y.; Guo Z. Optical Properties of Natural Small Molecules and Their Applications in Imaging and Nanomedicine. Adv. Drug Delivery Rev. 2021, 179, 113917 10.1016/j.addr.2021.113917. 34384827

[ref50] Cai X.-M.; Lin Y.; Li Y.; Chen X.; Wang Z.; Zhao X.; Huang S.; Zhao Z.; Tang B. Z. BioAIEgens Derived from Rosin: How Does Molecular Motion Affect Their Photophysical Processes in Solid State?. Nat. Commun. 2021, 12, 1773 10.1038/s41467-021-22061-y. 33741995 PMC7979920

[ref51] Wang J.; Huo F.; Zhang Y.; Yin C. Spiropyran Isomerization Triggering ESIPT for Visualization of pH Fluctuations During Oxidative Stress in Living Cells. Chin. Chem. Lett. 2023, 34, 107818 10.1016/j.cclet.2022.107818.

[ref52] Zhou L.; Jin Z.; Fan X.; Yao Y.; Chen Z.; Zhang W.; Qian J. Synthesis of 1,8-Naphthalimide-Based Fluorescent Nano-Probes and Their Application in pH Detection. Chin. Chem. Lett. 2018, 29, 1500–1502. 10.1016/j.cclet.2018.07.018.

[ref53] Wen Y.; Zhang W.; Liu T.; Huo F.; Yin C. Pinpoint Diagnostic Kit for Heat Stroke by Monitoring Lysosomal pH. Anal. Chem. 2017, 89, 11869–11874. 10.1021/acs.analchem.7b03612. 28992693

[ref54] Mj F.; Gw T.; Hb S.; Ge S.; Ma R.; Jr Cheeseman G.; Scalmani V.; Mennucci B.; Petersson G., Gaussian09, R. A. 1; Gaussian. Inc.: Wallingford CT, 2009, 121, 150 166.

[ref55] Wu Q.; Liu J.; Li Y.; Lee M. M.; Hu L.; Li Y.; Zhou P.; Wang D.; Tang B. Z. Janus Luminogens with Bended Intramolecular Charge Transfer: Toward Molecular Transistor and Brain Imaging. Matter 2021, 4, 3286–3300. 10.1016/j.matt.2021.08.002.

[ref56] Zheng J.; Fan R.; Wu H.; Yao H.; Yan Y.; Liu J.; Ran L.; Sun Z.; Yi L.; Dang L.; Gan P.; Zheng P.; Yang T.; Zhang Y.; Tang T.; Wang Y. Directed Self-Assembly of Herbal Small Molecules into Sustained Release Hydrogels for Treating Neural Inflammation. Nat. Commun. 2019, 10 (1), 1604 10.1038/s41467-019-09601-3. 30962431 PMC6453967

[ref57] Wu M.-Y.; Gu M.; Leung J.-K.; Li X.; Yuan Y.; Shen C.; Wang L.; Zhao E.; Chen S. A Membrane-Targeting Photosensitizer with Aggregation-Induced Emission Characteristics for Highly Efficient Photodynamic Combat of Human Coronaviruses. Small 2021, 17 (30), 2101770 10.1002/smll.202101770. 34190409 PMC8420407

[ref58] Wu M.; Leung J.; Kam C.; Chou T.; Wang D.; Feng S.; Chen S. A Near-Infrared AIE Probe for Super-Resolution Imaging and Nuclear Lipid Droplet Dynamic Study. Mater. Chem. Front. 2021, 5, 3043–3049. 10.1039/D0QM00914H.

[ref59] Dahllöf B.; Billström A.; Cabral F.; Hartley-Asp B. Estramustine Depolymerizes Microtubules by Binding to Tubulin. Cancer Res. 1993, 53 (19), 4573–4581. 8402630

[ref60] Inokuchi Y.; Nakajima Y.; Shimazawa M.; Kurita T.; Kubo M.; Saito A.; Sajiki H.; Kudo T.; Aihara M.; Imaizumi K.; Araie M.; et al. Effect of an Inducer of Bip, a Molecular Chaperone, on Endoplasmic Reticulum (ER) Stress-Induced Retinal Cell Death. Invest. Ophthalmol. Visual Sci. 2009, 50 (1), 334–344. 10.1167/iovs.08-2123. 18757512

[ref61] Zhao N.; Chen S.; Hong Y.; Tang B. Z. A Red Emitting Mitochondria-Targeted AIE Probe as an Indicator for Membrane Potential and Mouse Sperm Activity. Chem. Commun. 2015, 51, 13599–13602. 10.1039/C5CC04731E. 26264419

[ref62] Jia F.; Chibhabha F.; Yang Y.; Kuang Y.; Zhang Q.; Ullah S.; Liang Z.; Xie M.; Li F. Detection and Monitoring of the Neuroprotective Behavior of Curcumin Micelles Based on an AIEgen Probe. J. Mater. Chem. B 2021, 9, 731–745. 10.1039/D0TB02320E. 33315037

[ref63] Lo C. Y.-W.; Chen S.; Creed S. J.; Kang M.; Zhao N.; Tang B. Z.; Elgass K. D. Novel Super-Resolution Capable Mitochondrial Probe, Mitored AIE, Enables Assessment of Real-Time Molecular Mitochondrial Dynamics. Sci. Rep. 2016, 6 (1), 30855 10.1038/srep30855. 27492961 PMC4974624

[ref64] Porat-Shliom N.; Chen Y.; Tora M.; Shitara A.; Masedunskas A.; Weigert R. In Vivo Tissue-Wide Synchronization of Mitochondrial Metabolic Oscillations. Cell Rep. 2014, 9, 514–521. 10.1016/j.celrep.2014.09.022. 25373899 PMC4223640

[ref65] Miguel A. A.; Cortassa S.; O’Rourke B. Percolation and Criticality in a Mitochondrial Network. Proc. Natl. Acad. Sci. U. S. A. 2004, 101, 4447–4454. 10.1073/pnas.0307156101. 15070738 PMC384767

[ref66] Gerencser A. A.; Adam-Vizi V. Mitochondrial Ca^2+^ Dynamics Reveals Limited Intramitochondrial Ca^2+^ Diffusion. Biophys. J. 2005, 88, 698–714. 10.1529/biophysj.104.050062. 15501949 PMC1305047

[ref67] Leanza L.; Romio M.; Becker K. A.; Azzolini M.; Trentin L.; Manago A.; Venturini E.; Zaccagnino A.; Mattarei A.; Carraretto L.; Urbani A.; et al. Direct Pharmacological Targeting of a Mitochondrial Ion Channel Selectively Kills Tumor Cells in Vivo. Cancer Cell 2017, 31 (4), 516–531.e10. 10.1016/j.ccell.2017.03.003. 28399409

[ref68] Liu Z.; Xiao T. S. Partners with a Killer: Metabolic Signaling Promotes Inflammatory Cell Death. Cell 2021, 184, 4374–4376. 10.1016/j.cell.2021.07.036. 34416144 PMC9847695

