# Supplementary material for: Fluorescent Nanocable as a Biomedical Tool: Intracellular Self-Assembly Formed by a Natural Product Interconnects and Synchronizes Mitochondria
Source: ACS Nano. 2024 Jul 31;18(32):21447–58. doi: 10.1021/acsnano.4c06186 (PMC11328177; doi:10.1021/acsnano.4c06186)
Supplement: Supplementary file 1 — nn4c06186_si_001.pdf [file nn4c06186_si_001.pdf]

Supporting Information

Fluorescent Nanocable as a Biomedical Tool: Intracellular Self-assembly Formed by a Natural Product Interconnects and Synchronizes Mitochondria

Xueqian Zhao,[1,2†] Fei Wang,[1,2†] Chuen Kam,[1,2] Ming-Yu Wu,[2] Jianyu Zhang,[3] Changhuo Xu,[4] Kai Bao,[5] Qiyuan He,[5] Ruquan Ye,[5] Ben Zhong Tang,[3,6] Sijie Chen[1,2]*

1. School of Life Sciences, The Chinese University of Hong Kong, Hong Kong 999077, China

2. Ming Wai Lau Centre for Reparative Medicine, Karolinska Institutet, Hong Kong 999077, China; Ming Wai Lau Centre for Reparative Medicine, Department of Neuroscience, Karolinska Institutet, Stockholm, 17177, Sweden

3. Department of Chemistry, Hong Kong Branch of Chinese National Engineering Research Center for Tissue Restoration and Reconstruction, and Guangdong-Hong Kong-Macau Joint Laboratory of Optoelectronic and Magnetic Functional Materials, The Hong Kong University of Science and Technology, Hong Kong 999077, China

4. Ministry of Education Frontiers Science Center for Precision Oncology, Faculty of Health Sciences, University of Macau, Macao 999078, China

5. Department of Materials Science and Engineering, Department of Chemistry, City University of Hong Kong, Hong Kong 999077, China

6. School of Science and Engineering, Shenzhen Institute of Aggregate Science and Technology, The Chinese University of Hong Kong, Shenzhen (CUHK-Shenzhen), Guangdong 518172, China

*Corresponding Author: Sijie Chen, sijie.chen@cuhk.edu.hk

†These authors contributed equally to this work.

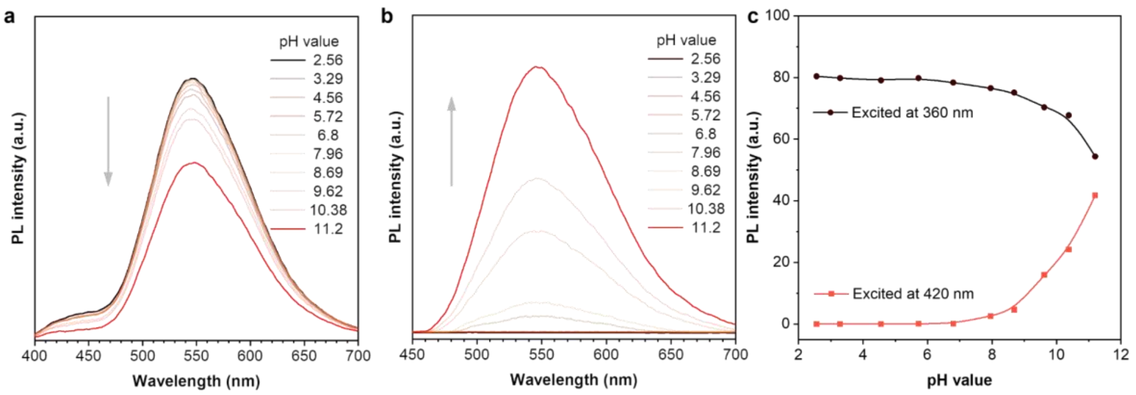

Figure S1. Emission spectra of LBT (10 μM) in the B-R buffer solution at different pH values (2.56 – 11.2) excited at (**a**) 360 nm, (**b**) 420 nm. (**c**) The plot of emission intensity at 550 nm versus pH value.

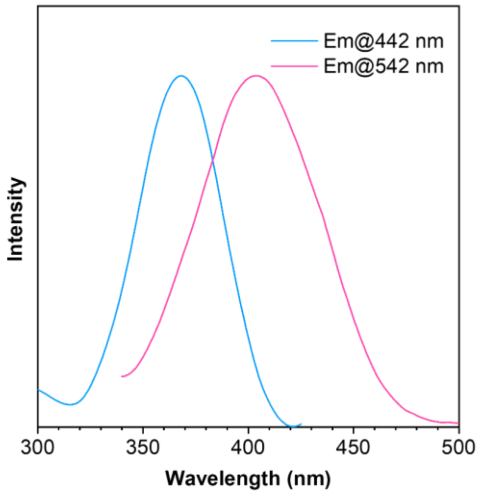

Figure S2. Normalized excitation spectra of LBT in aqueous solutions at room temperature.

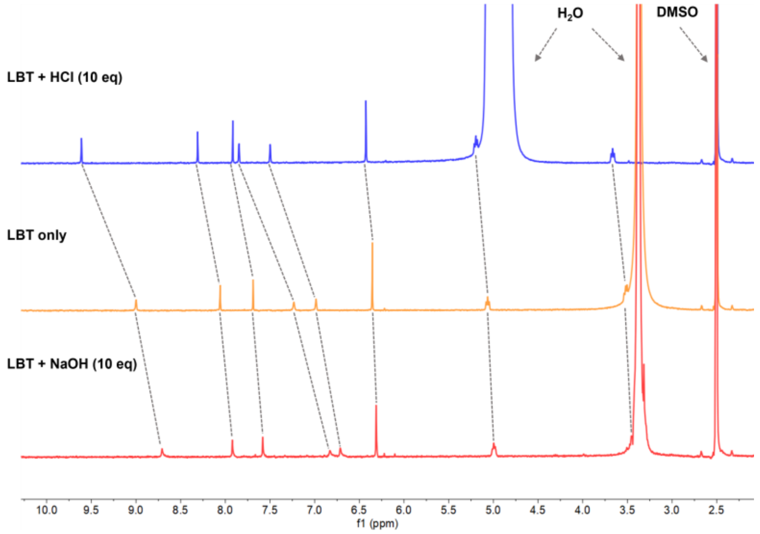

Figure S3. ^{1}H NMR spectra of LBT treated with HCl or NaOH (400 MHz, DMSO-d_6).

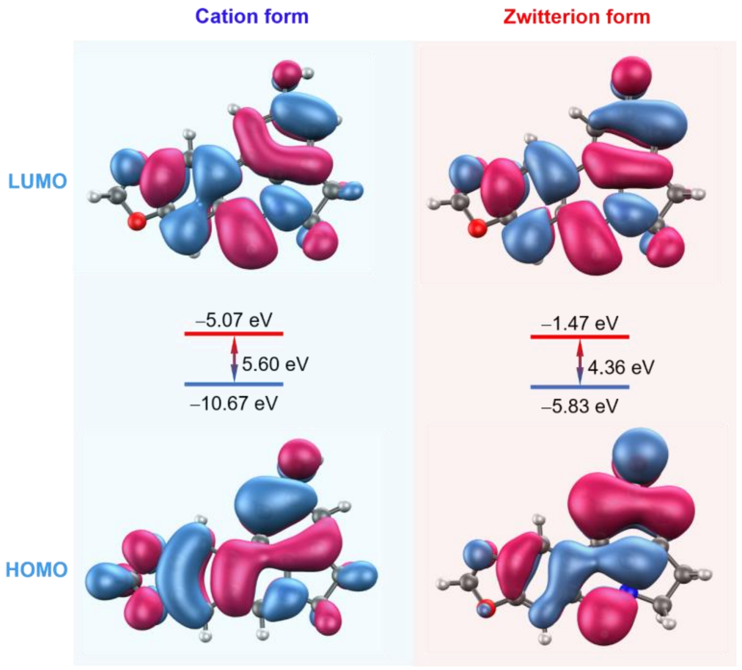

Figure S4. HOMO and LUMO plots and corresponding energy gaps from DFT calculations in the gas state. All the above quantum chemical calculations were carried out using the Gaussian 09 program.[1]

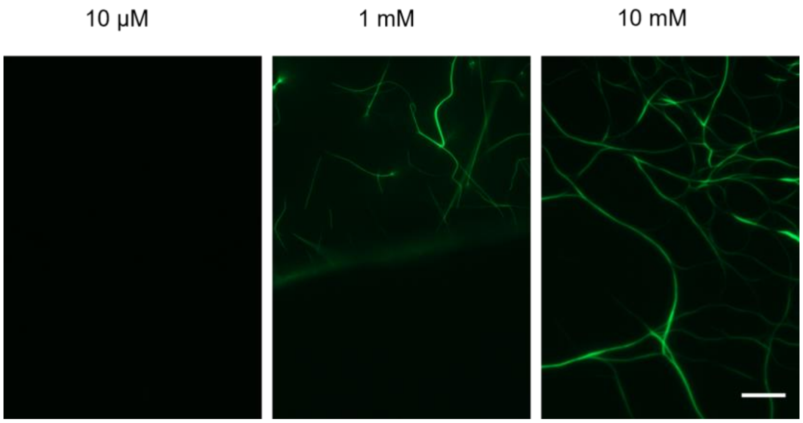

Figure S5. Fluorescence imaging of self-assembly LBT nanofibers with different concentrations on the surface of the glass sheet excited by a FITC light source (λ_{ex} = 465–495 nm, λ_{em} = 512–558 nm). Scale bar = 20 µm.

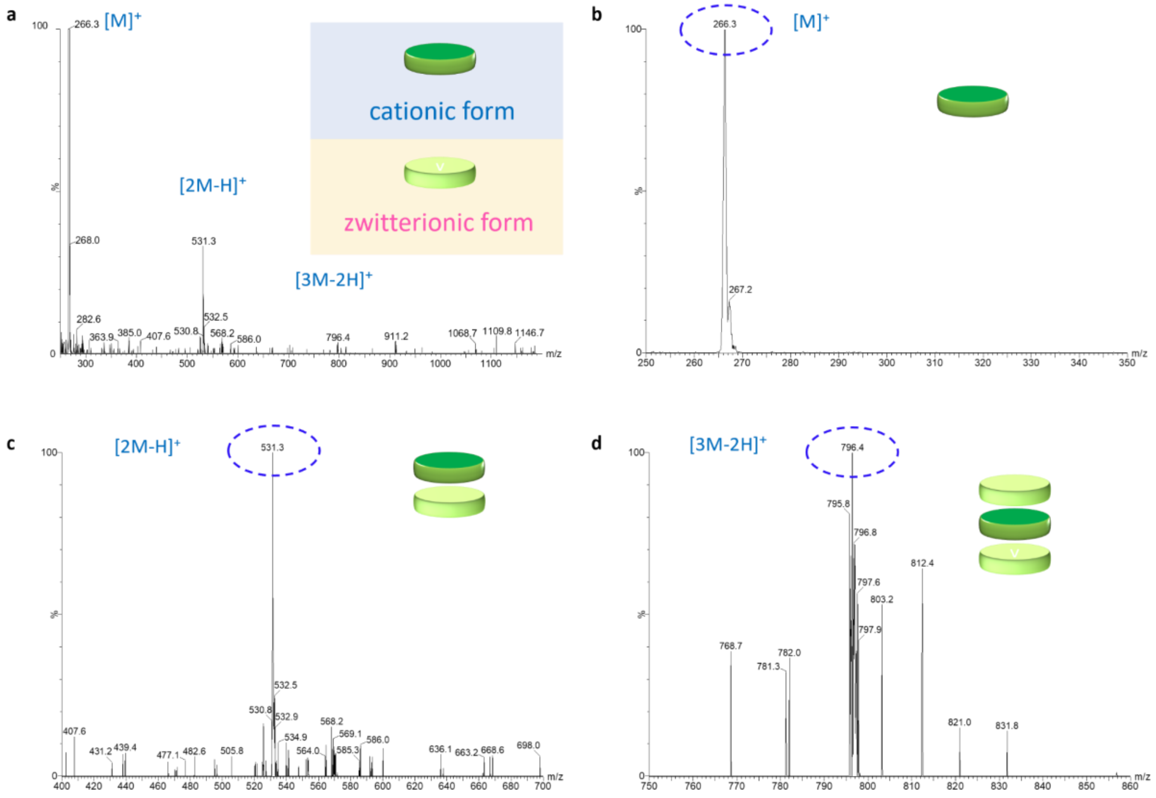

Figure S6. (a-d) ESI-MS calcd. for C16H12NO3$^+$, [M]$^+$ m/z =266.1, found 266.3; C32H23N2O6$^+$, [2M-H]$^+$ m/z = 531.1, found 531.3; C48H34N3O9$^+$, [3M-2H]$^+$ 796.2, found 796.4.

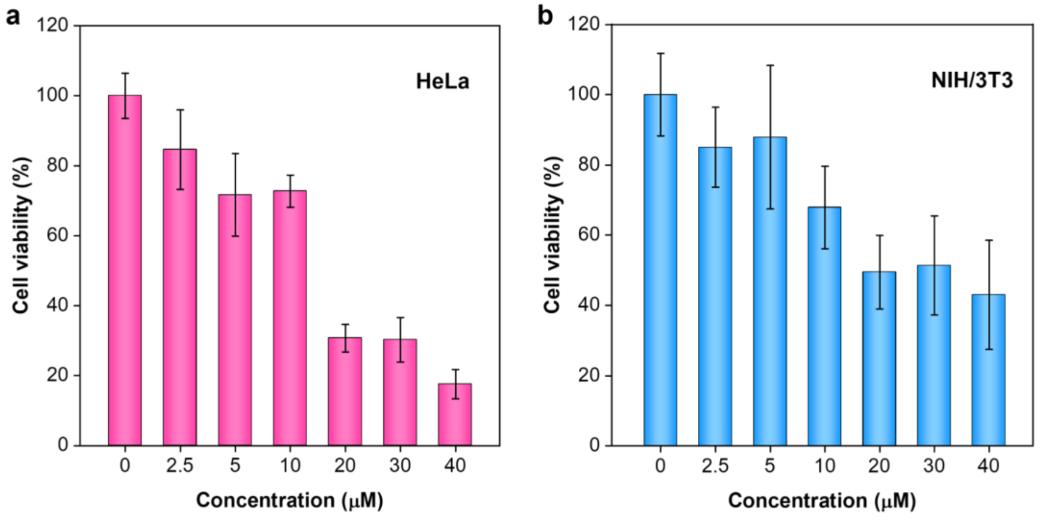

Figure S7. The CCK-8 assay was performed in (**a**) HeLa cells and (**b**) NIH/3T3 cells after incubation with different concentrations of LBT for 24 hours.

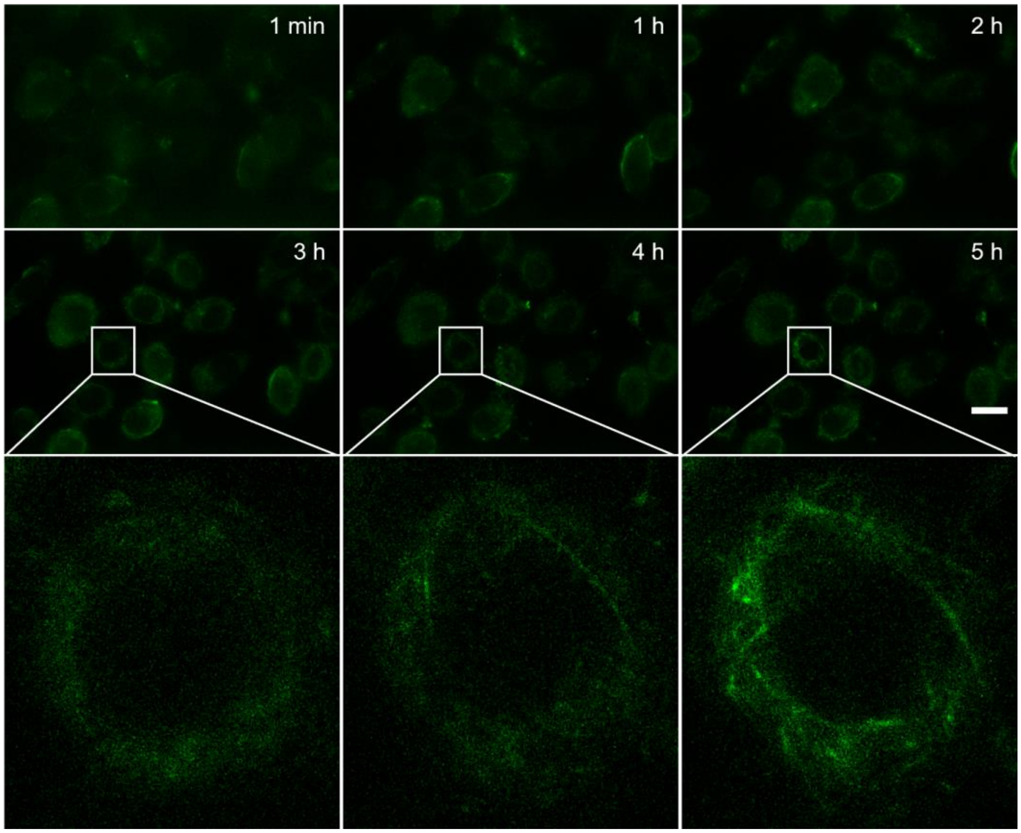

Figure S8. Time-dependent monitoring of cellular uptake and distribution of LBT (10 μM) in HeLa cells at 37 °C (λ_{ex} = 465–495 nm, λ_{em} = 512–558 nm). Scale bar = 20 μm.

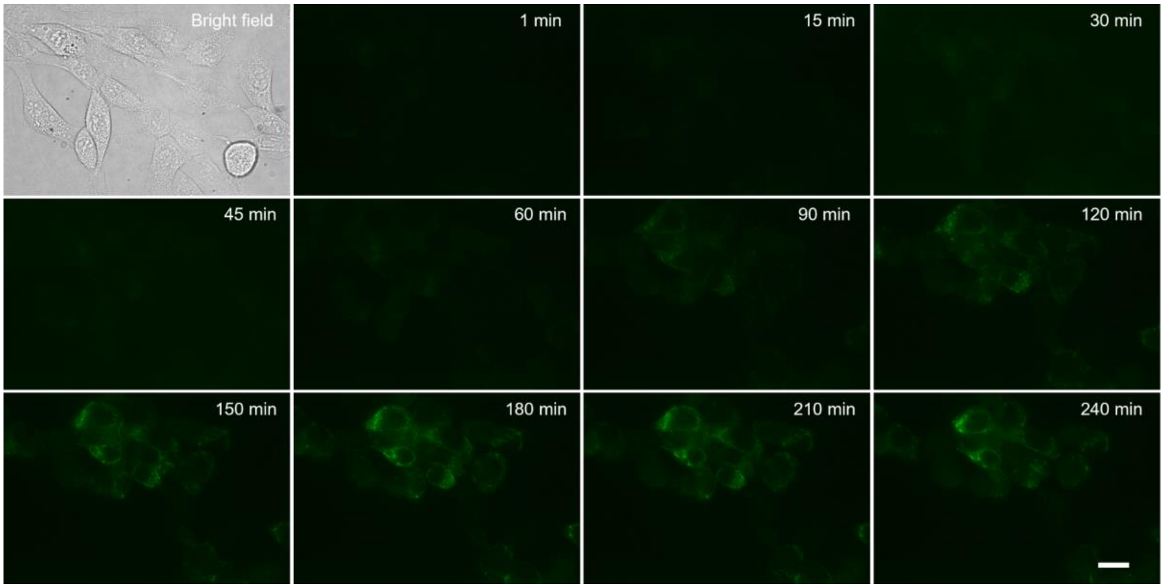

Figure S9. Time-dependent monitoring of cellular uptake and distribution of LBT (10 μM) in NIH/3T3 cells 37 °C (λ_{ex} = 465–495 nm, λ_{em} = 512–558 nm). Scale bar = 20 μm.

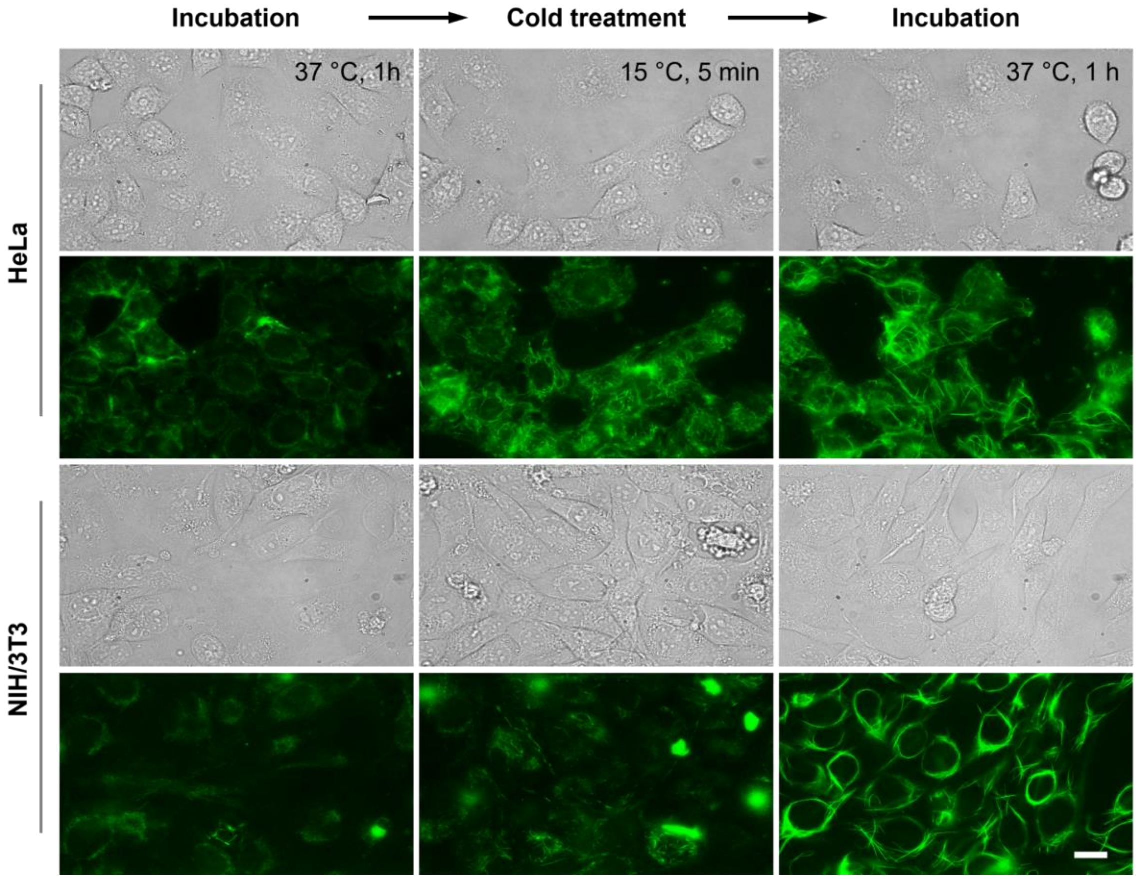

Figure S10. Staining images of living HeLa and NIH/3T3 cells incubated with LBT (10 μM) (λ_{ex} = 465–495 nm, λ_{em} = 512–558 nm). Scale bar = 20 μm.

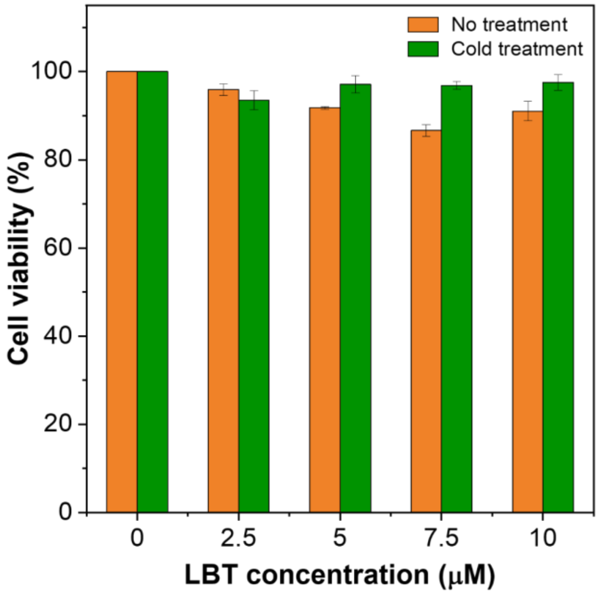

Figure S11. The CCK-8 assay was performed in HeLa cells after incubation with different concentrations of LBT for 2 hours.

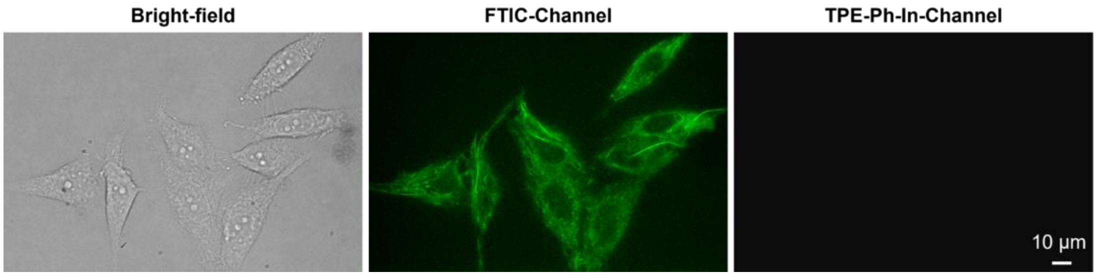

Figure S12. The fluorescence images of HeLa cells. Cells were incubated with LBT (10 μM) only for 2 h followed by the cold treatment. Green fluorescence is from LBT (λ_{ex} = 465–495 nm, λ_{em} = 512–558 nm); fluorescence signal in TPE-Ph-In channel is also collected (λ_{ex} = 509–519 nm, Emission was collected using a long-pass filter with a cutoff wavelength of 590 nm). Scale bar = 10 μm.

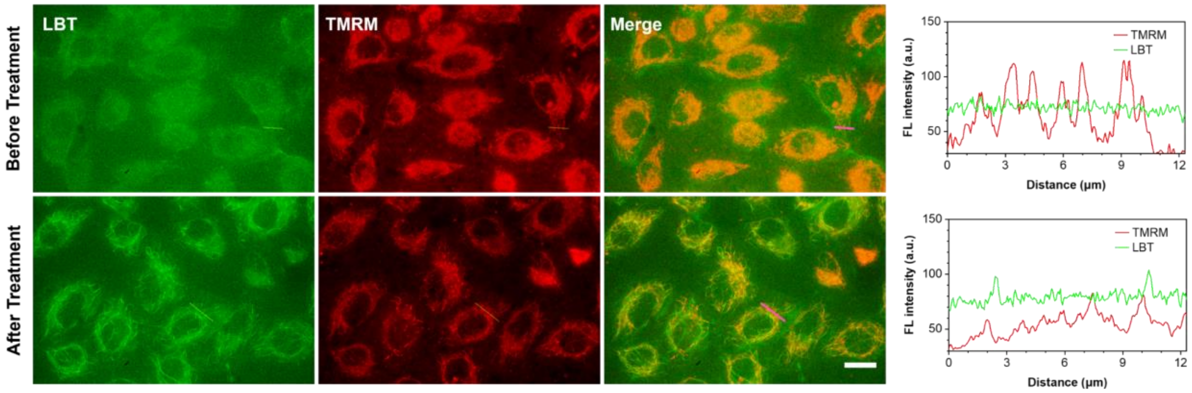

Figure S13. The fluorescence images of living HeLa cells. Cells were incubated with LBT (10 μM) and TMRM (20 nM) in for 1 h (37 °C) followed by the 5 min cold treatment (15 °C). The corresponding fluorescence intensity correlation curve between LBT and TMRM at the solid white line was shown. Green fluorescence is from LBT (λ_{ex} = 465 nm – 495 nm, λ_{em} = 512 nm – 558 nm); red fluorescence is from TMRM (λ_{ex} = 509–519 nm, Emission was collected using a long-pass filter with a cutoff wavelength of 590 nm). Scale bar = 20 μm.

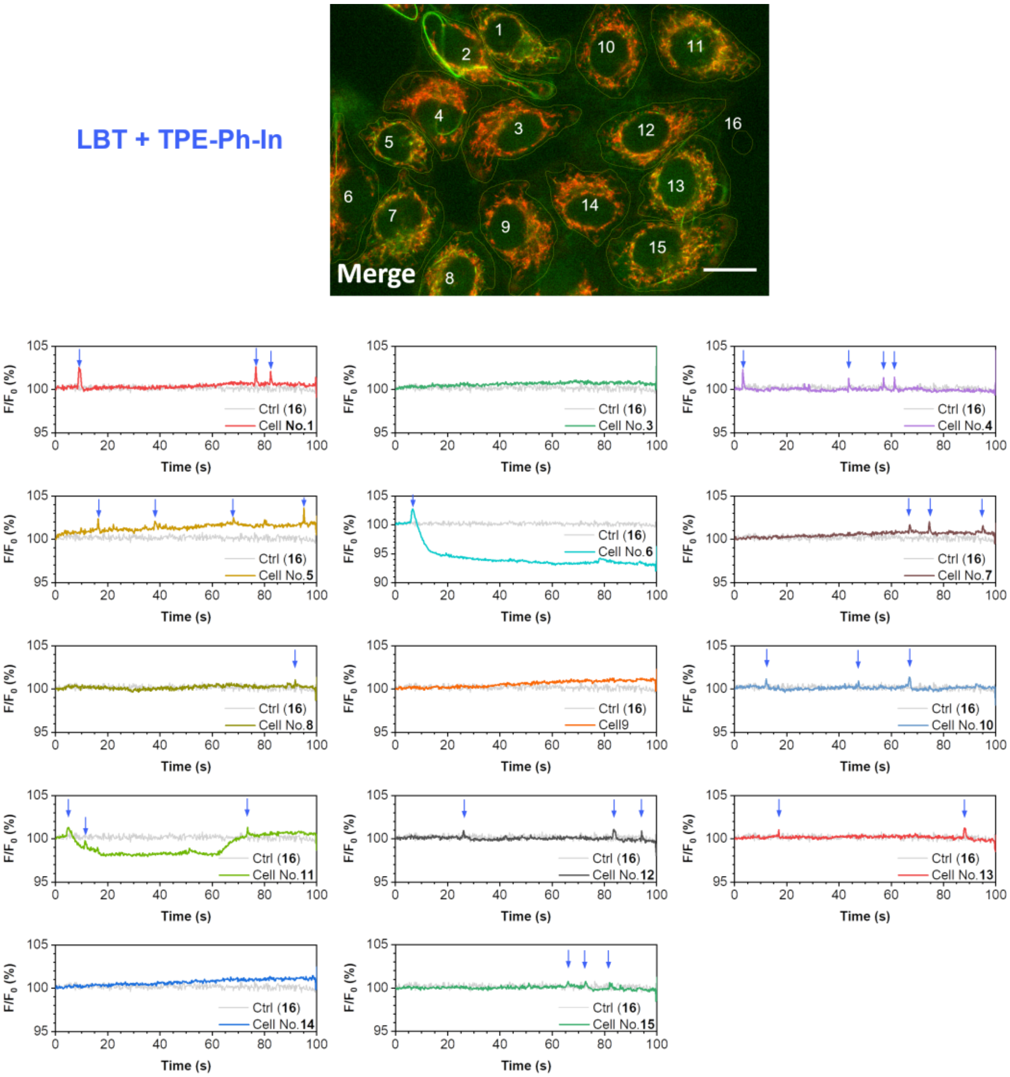

Figure S14. The fluorescence images of living HeLa cells. Cells were incubated with LBT (10 μM) and TPE-Ph-In (5 μM) for 2 hours followed by cold treatment. Each cell was picked and labeled as a fluorescent ROI. Time course analysis of TPE-Ph-In fluorescence intensities over 100 seconds was shown. No. 16 represents the FL intensity changes of the background. Green fluorescence is from LBT (λ_{ex} = 465–495 nm, λ_{em} = 512–558 nm); red fluorescence is from TPE-Ph-In (λ_{ex} = 509–519 nm, Emission was collected using a long-pass filter with a cutoff wavelength of 590 nm). Scale bar = 20 μm. F and F_0 represent the real-time and initial fluorescence intensities, respectively.

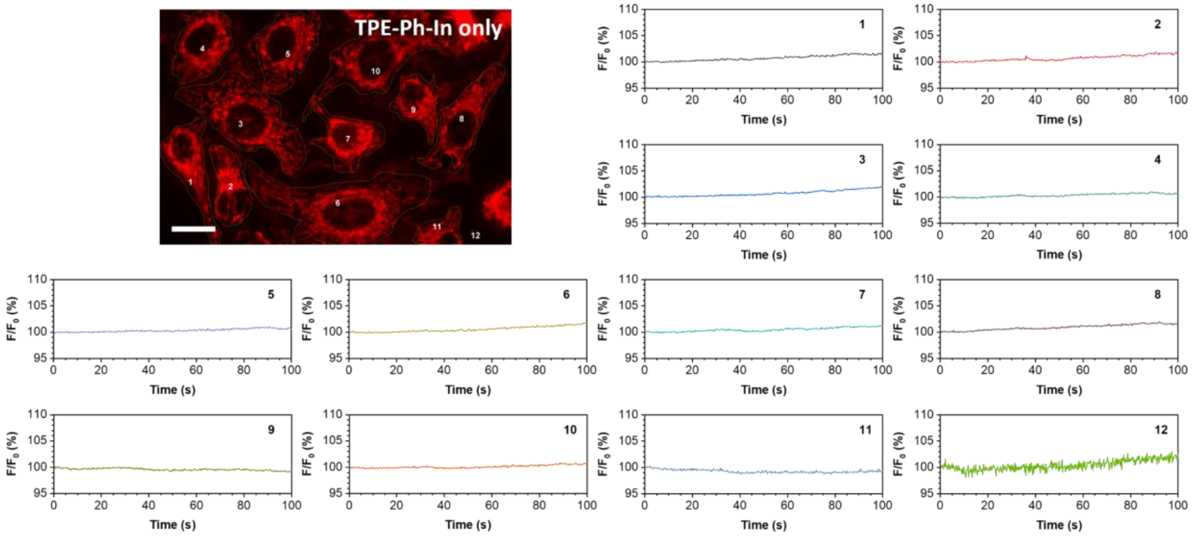

Figure S15. The fluorescence images of living HeLa cells. Cells were incubated with TPE-Ph-In (5 μM) only for 2 hours followed by the cold treatment. Each cell was picked and labeled as a fluorescent ROI. Time course analysis of TPE-Ph-In fluorescence intensities over 100 seconds was shown. No. 12 represents the FL intensity changes of the background. Red fluorescence is from TPE-Ph-In (λ_{ex} = 509–519 nm, Emission was collected using a long-pass filter with a cutoff wavelength of 590 nm). Scale bar = 20 μm. No spontaneous $\Delta\Psi_m$ fluctuation was observed at the cellular level. F and F_0 represent the real-time and initial fluorescence intensities, respectively.

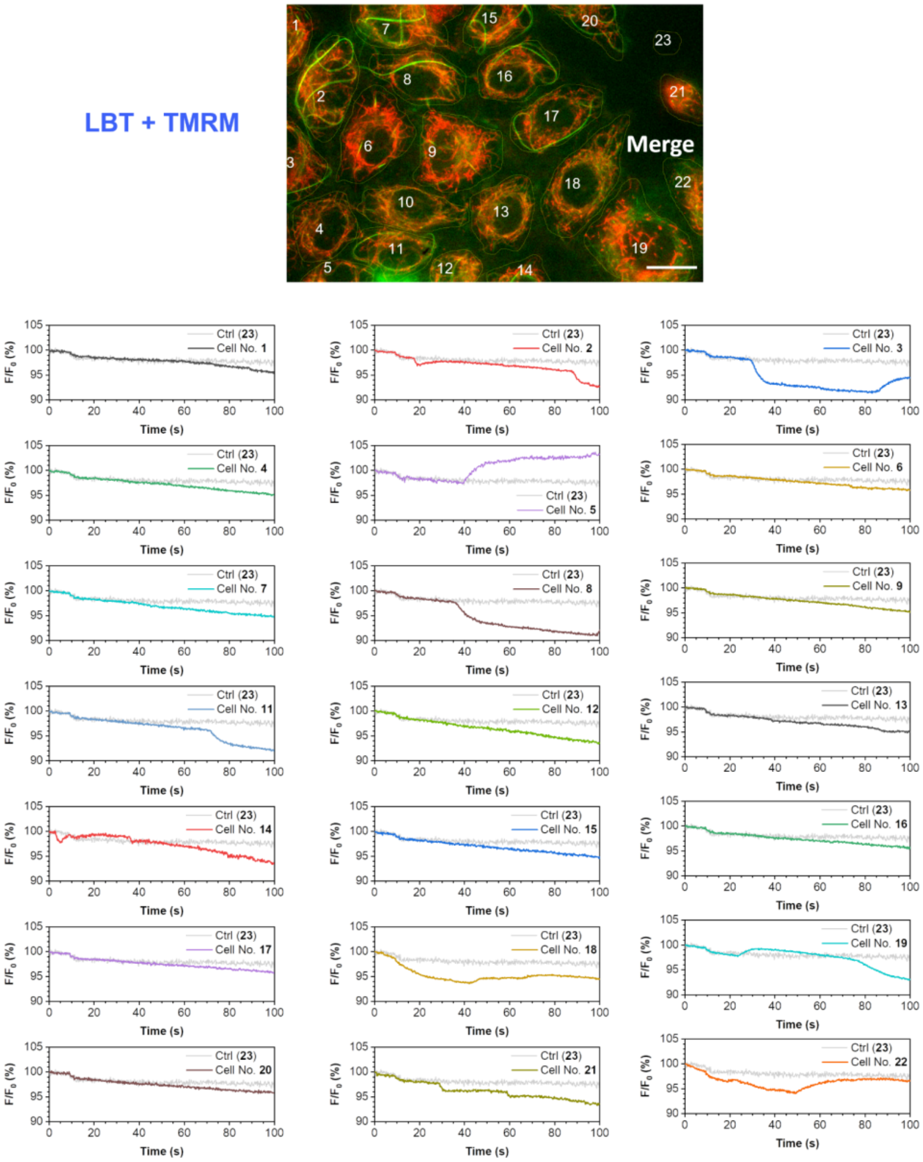

Figure S16. The fluorescence images of living HeLa cells. Cells were incubated with LBT (10 μM) and TMRM (20 nM) for 2 hours followed by cold treatment. Each cell was picked and labeled as a fluorescent ROI. Time course analysis of TMRM fluorescence intensities over 100 seconds was shown. No. 23 represents the FL intensity changes of the background. Green fluorescence is from LBT (λ_{ex} = 465–495 nm, λ_{em} = 512–558 nm); red fluorescence is from TMRM (λ_{ex} = 509–519 nm, Emission was collected using a long-pass filter with a cutoff wavelength of 590 nm). Scale bar = 20 μm. F and F_0 represent the real-time and initial fluorescence intensities, respectively.

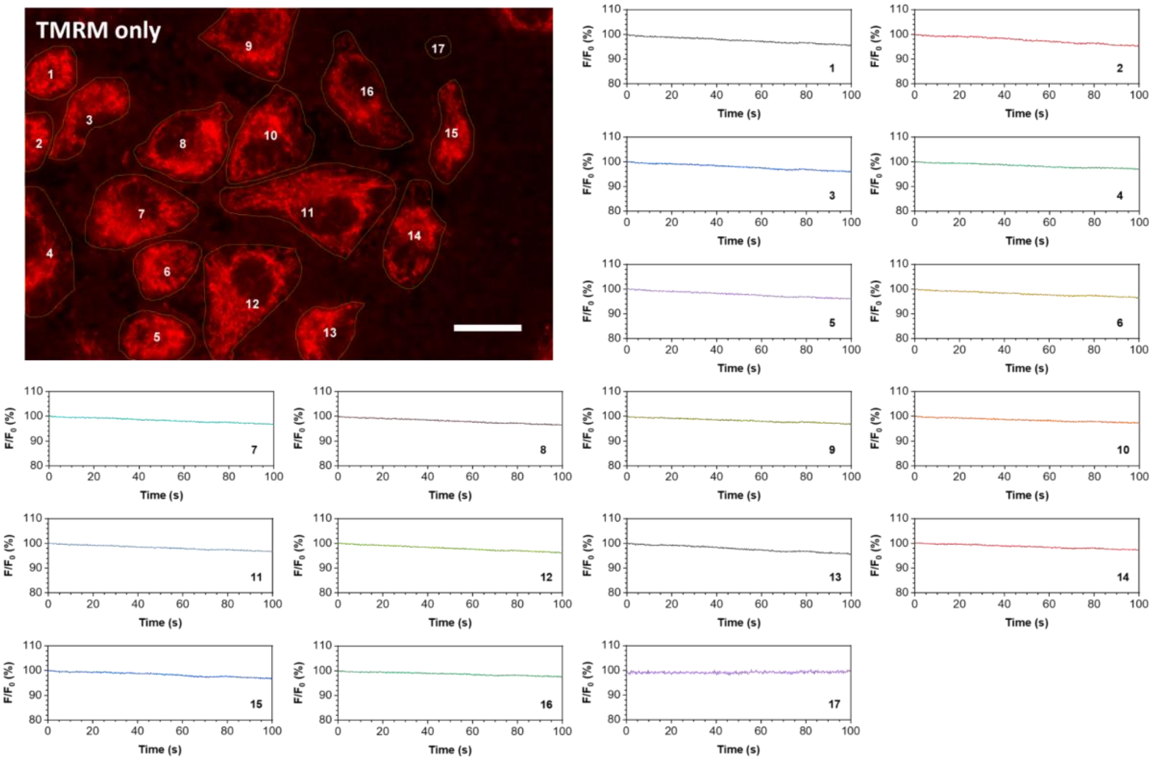

Figure S17. The fluorescence images of living HeLa cells. Cells were incubated with TMRM (20 nM) only for 2 hours followed by cold treatment. Each cell was picked and labeled as a fluorescent ROI. Time course analysis of TMRM fluorescence intensities over 100 seconds was shown. No. 17 represents the FL intensity changes of the background. Red fluorescence is from TMRM (λ_{ex} = 509–519 nm, Emission was collected using a long-pass filter with a cutoff wavelength of 590 nm). Scale bars = 20 μm. No spontaneous $\Delta\Psi_m$ fluctuation was observed at the cellular level. F and F_0 represent the real-time and initial fluorescence intensities, respectively.

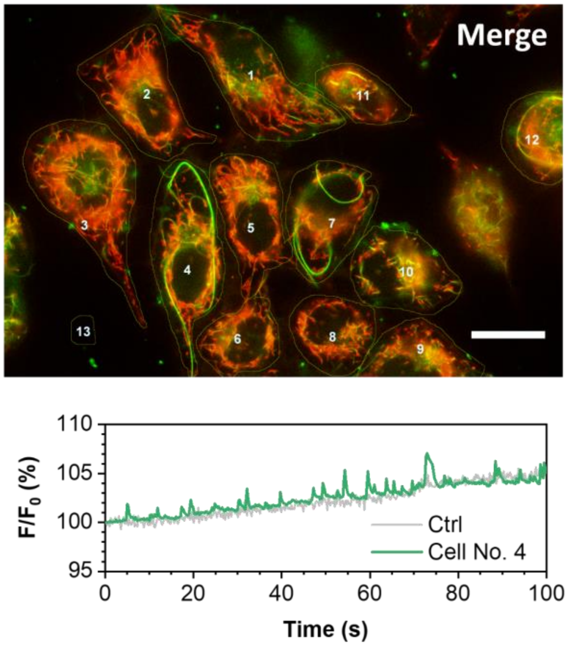

Figure S18. The fluorescence images of living HeLa cells. Cells were incubated with LBT (10μM) and TPE-Ph-In (5 μM) for 2 hours followed by cold treatment. Each cell was picked and labeled as a fluorescent ROI. Time course analysis of TPE-Ph-In fluorescence intensities over the 100 seconds was shown. No. 13 (Ctrl) represents the FL intensity changes of the background. Green fluorescence is from LBT (λ_{ex} = 465–495 nm, λ_{em} = 512–558 nm); red fluorescence is from TPE-Ph-In (λ_{ex} = 509–519 nm, Emission was collected using a long-pass filter with a cutoff wavelength of 590 nm). Scale bars = 20 μm. F and F_0 represent the real-time and initial fluorescence intensities, respectively.

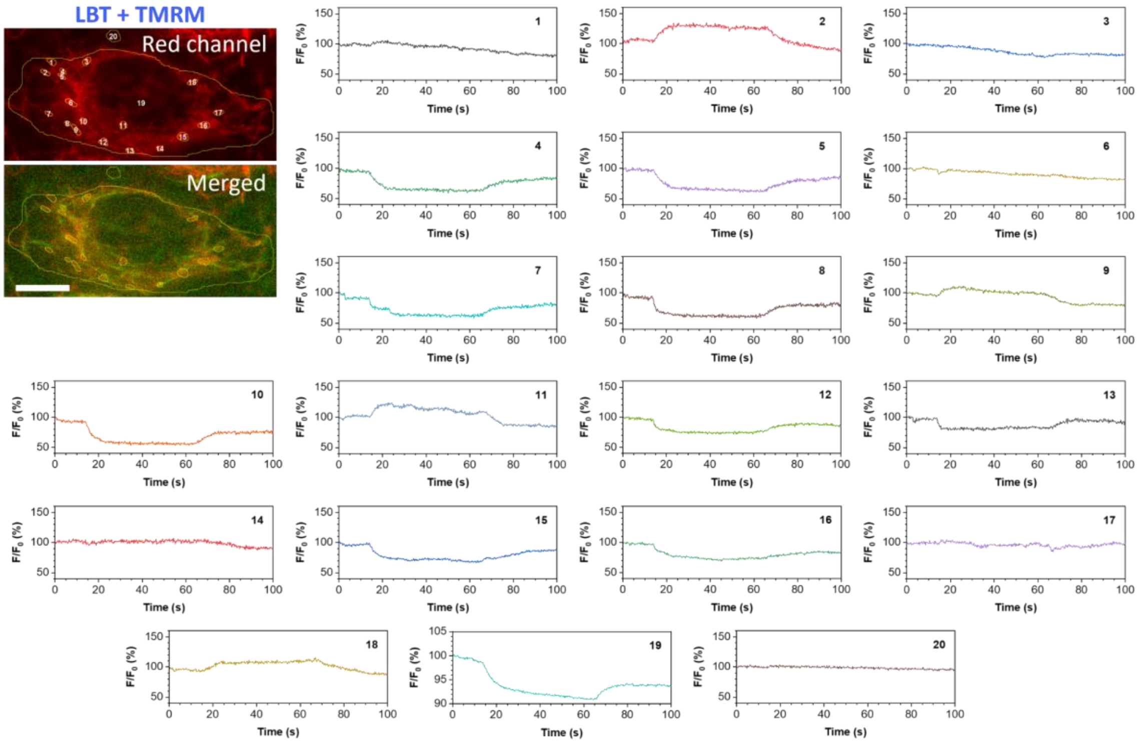

Figure S19. Fluorescence images of the HeLa cell No. 10 from Figure **S16**. Representative mitochondria were picked and labeled as a fluorescent ROI and the fluorescence intensity over the course (100 seconds) was recorded. No. 19 represents the FL intensity changes of cell. No. 20 represents the FL intensity changes of the background. Green fluorescence is from LBT (λ_{ex} = 465–495 nm, λ_{em} = 512–558 nm); red fluorescence is from TMRM (λ_{ex} = 509–519 nm, Emission was collected using a long-pass filter with a cutoff wavelength of 590 nm). Scale bar = 5 µm. F and F_0 represent the real-time and initial fluorescence intensities, respectively.

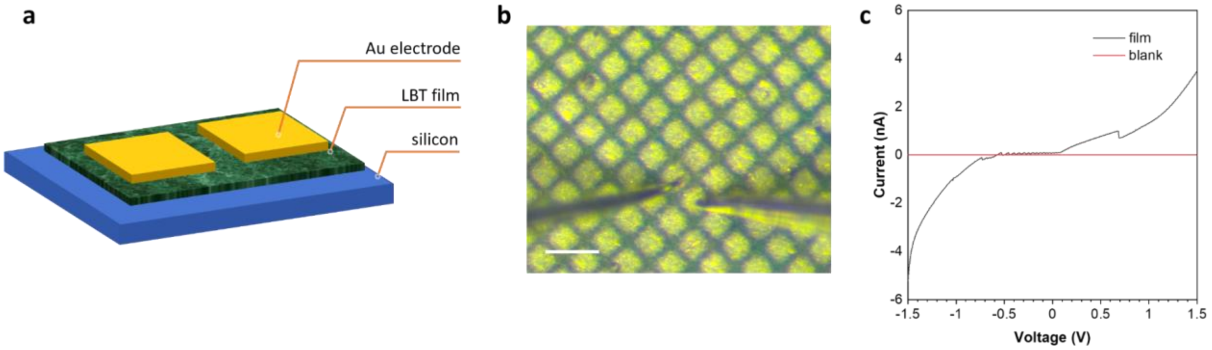

Figure S20. (**a**) Schematic illustration of the top electrode device. (**b**) Optical microscope images of the device. (**c**) Current-voltage characteristics of the device. Scale bar = 100 μM.

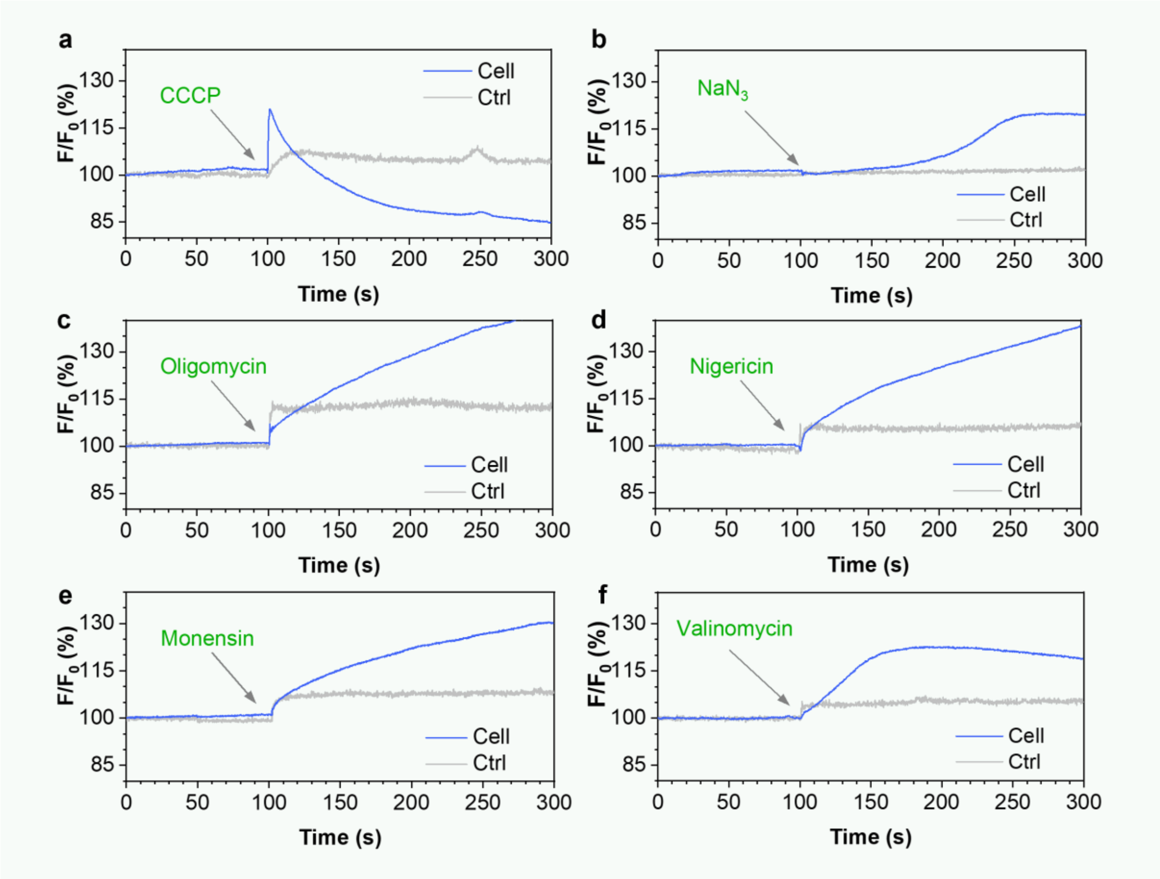

Figure S21. Fluorescence intensity (red channel) changes of TPE-Ph-In stained living HeLa cells before and after the treatment of different drugs. Ctrl represents the fluorescence intensity of blank area. F and F_0 represent the real-time and initial fluorescence intensities, respectively.

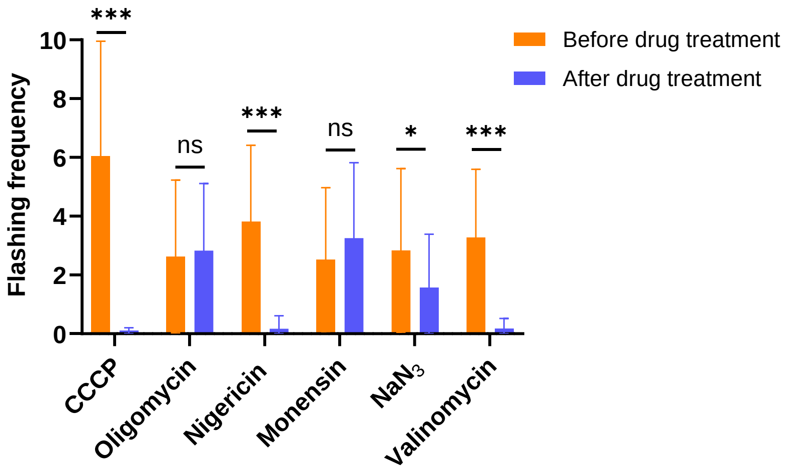

Figure S22. Effects of various drugs on $\Delta\Psi_m$ fluctuation frequency within per 100 s of HeLa Cell. The data were analyzed and expressed in the mean ± SD. Differences were considered significant at $p < 0.05$ (*), $p < 0.01$ (**) and $p < 0.001$ (***), ns was considered nonsignificant difference (two-tailed student's t-test). The number of HeLa cells analyzed for each drug treatment is as follows: CCCP (n = 23), Nigericin (n = 38), NaN_3 (n = 37), Oligomycin (n = 43), Monensin (n = 40), and Valinomycin (n = 61). Each experiment was repeated at least three times to ensure the reliability of the results.

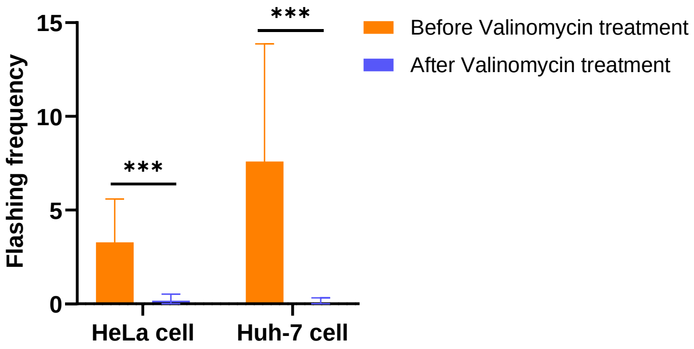

Figure S23. Effects of valinomycin on $\Delta\Psi_m$ fluctuation frequency within per 100 s of HeLa and Huh-7 cells. The data were analyzed and expressed in the mean ± SD. Differences were considered significant at $p < 0.05$ (*), $p < 0.01$ (**) and $p < 0.001$ (***), ns was considered nonsignificant difference (two-tailed student's t-test). The number of HeLa and Huh-7 cells analyzed is as follows: HeLa cell (n = 61), Huh-7 cell (n = 15). Each experiment was repeated at least three times to ensure the reliability of the results.

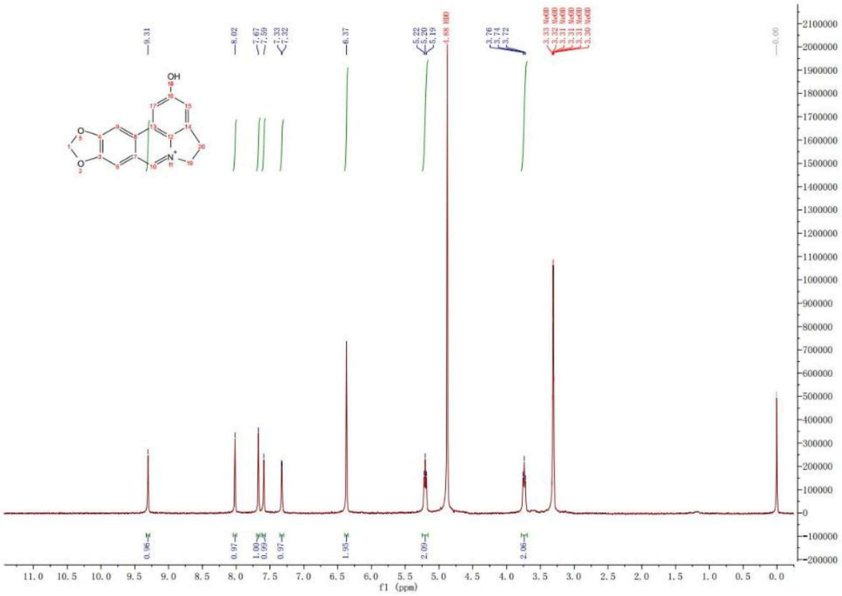

Figure S24. ^{1}H NMR spectra of LBT. (400 MHz, D_2O and CD_3OD).

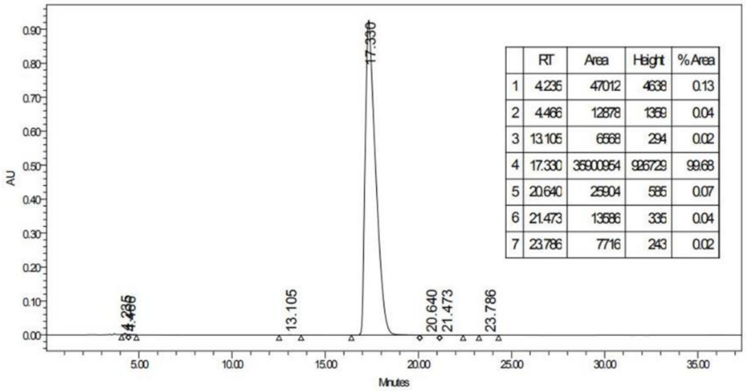

Figure S25. High-performance liquid chromatography (HPLC) of LBT. Mobile phase: acetonitrile: triethylamine phosphate (pH=3) = 10:90; detection wavelength: 260 nm.

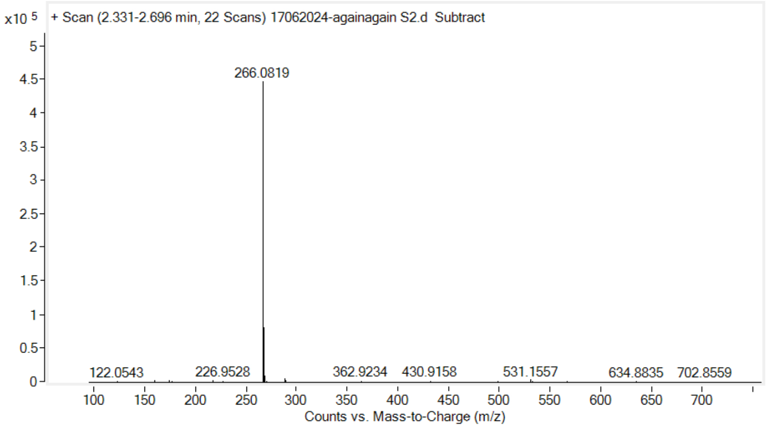

Figure S26. Electrospray ionization mass spectrometry (ESI-MS) analysis of LBT.

Table S1. Crystal data and structure refinement for the cationic form of LBT.[2]

Identification code	LBT
Empirical formula	C16 H14 Cl N O4
Formula weight	319.73
Temperature	298(2) K
Wavelength	0.71073 Å
Crystal system	Monoclinic
Space group	$P2_1/n$
	a = 11.0314(12) Å, a= 90°.
Unit cell dimensions	b = 7.1245(7) Å, b= 95.154(3)°.
	c = 18.1923(18) Å, g = 90°.
Volume	1424.0(3) Å^3
Z	4
Density (calculated)	1.491 g/cm^3
Absorption coefficient	0.287 mm^{-1}
F(000)	664
Crystal size	0.300 x 0.100 x 0.100 mm^3
Theta range for data collection	3.072 to 25.244°.
Index ranges	-13<=h<=13, -8<=k<=8, -21<=l<=21
Reflections collected	18895
Independent reflections	2577 [R(int) = 0.0389]
Completeness to theta = 25.242°	99.7 %
Absorption correction	multi-scan
Max. and min. transmission	0.7456 and 0.6969
Refinement method	Full-matrix least-squares on F^2
Data/restraints/parameters	2577 / 3 / 207
Goodness-of-fit on F^2	1.057
Final R indices [I>2sigma(I)]	R1 = 0.0475, wR2 = 0.1132
R indices (all data)	R1 = 0.0637, wR2 = 0.1221
Extinction coefficient	n/a
Largest diff. peak and hole	0.340 and -0.333 e.Å^{-3}

Table S2 Statistics and reproducibility of cell images.

Experimental description	Cold treatment	Repetition times	Vides	Number of cells
Figure **3** (confocal image)		3		
Figure **4** (co-localization)		3		
TPE-Ph-In (10 μM) only	N	3	10	124
TPE-Ph-In (10 μM) only	Y	3	8	91
LBT and TPE-Ph-In (10 μM)	N	4	17	234
LBT and TPE-Ph-In (10 μM)	Y	3	11	143
LBT and TPE-Ph-In (2.5 μM)	N	1	4	57
LBT and TPE-Ph-In (2.5 μM)	Y	1	4	58
LBT and TPE-Ph-In (5 μM)	N	1	6	74
LBT and TPE-Ph-In (5 μM)	Y	1	7	98
LBT and TMRM (20 nM)	N	3	15	
LBT and TMRM (20 nM)	Y	3	18	

References

1. Frisch, M. J.; Trucks, G. W.; Schlegel, H. B.; Scuseria, G. E.; Robb, M. A.; Cheeseman, J. R.; Scalmani, G.; Barone, V.; Petersson, G. A.; Nakatsuji, H.; Li, X.; Caricato, M.; Marenich, A.; Bloino, J.; Janesko, B. G.; Gomperts, R.; Mennucci, B.; Hratchian, H. P.; Ortiz, J. V.; Izmaylov, A. F. et al. Gaussian 09. **2016**, *Revision A.02.*, Gaussian, Inc., Wallingford CT.

2. Xueqian ZHAO CCDC 2202095: Experimental Crystal Structure Determination, 2022, DOI: 10.5517/ccdc.csd.cc2cxgb3
